# Thiol-mediated uptake of phosphorothioate liposomes, visualized with fluorescent flippers

**DOI:** 10.1039/d5sc05796e

**Published:** 2025-09-29

**Authors:** Jules Bouffard, Felix Bayard, Naomi Sakai, Stefan Matile

**Affiliations:** a Department of Organic Chemistry, University of Geneva Geneva Switzerland stefan.matile@unige.ch www.unige.ch/sciences/chiorg/matile/ +41 22 379 6523; b National Centre of Competence in Research (NCCR) Molecular Systems Engineering Basel BPR 1095 Switzerland

## Abstract

Liposomes made from phosphorothioate lipids are shown to penetrate cells better and differently than conventional phosphodiester liposomes. DSP_S_C phosphorothioate liposomes are synthesized, characterized and labeled with either internal doxorubicin or membrane-bound flippers. Inhibition experiments reveal that their penetration of HK cells is independent of endocytosis and occurs by thiol-mediated uptake (TMU). Dynamic covalent exchange with phosphorothioate sulfurs as pseudo-thiolates is confirmed and explored to modify liposomes and activate TMU. Mechanosensitive flipper probes and colocalization experiments reveal that phosphorothioate liposomes cross the plasma membrane in intact form with negligible endocytosis and little fusion. In the cytosol, fast-emitting flipper probes and non-released doxorubicin in punctate objects that partially co-localize with lipid droplets but not lysosomes suggest that the liposomes apparently stay at least partially intact and incorporate disorganizing lipid components from lipid droplets. In clear contrast, conventional DSPC liposomes bind to the cell surface in intact form and neither fuse nor cross the plasma membrane. These results support and translate recent insights from cell-penetrating oligonucleotides to phosphorothioate lipids, highlight the importance of understanding the dynamic covalent chemistry of phosphorothioates, and identify flipper dendrons as promising tools to elucidate liposomal delivery.

## Abstract 2

The replacement of one oxygen by one sulfur atom in phospholipids affords phosphorothioate liposomes that enter cells by thiol-mediated uptake, with negligible contributions from endocytosis and fusion.

## Graphical abstract

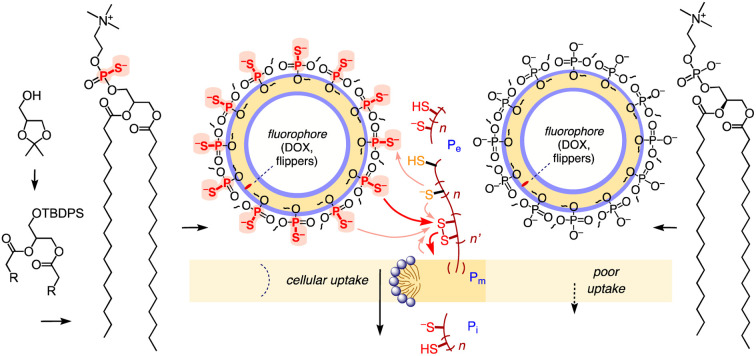

In oligonucleotide phosphorothioates, one oxygen per phosphodiester in native oligonucleotides is replaced by a sulfur.^1–4^ This single-atom substitution is the basis of approved antisense therapeutics that are used in practice. This success originates in part from the ability of oligonucleotide phosphorothioates to penetrate cells efficiently, while oligonucleotide phosphodiesters cannot. Oligonucleotide phosphorothioates appear as the oligonucleotide counterpart of arginine-rich cell-penetrating peptides (CPPs) in peptide chemistry, and the mechanism by which these polycations enter cells has fascinated the community for more than three decades.^5–9^ Although the question of how oligonucleotide phosphorothioates penetrate cells has attracted similar interest, their mode of action has remained unclear.^1–4^ How can the replacement of an oxygen atom by a sulfur cause this fundamental change?

In 2021, based on an inhibitor screen, we have suggested that the cell-penetrating nature of oligonucleotide phosphorothioates could originate from thiol-mediated uptake (TMU).^10^ TMU refers to the emergence of cell-penetrating activity in the substrate upon attachment of a motif capable of repeated dynamic covalent exchange with thiols and disulfides of cellular proteins.^5,11–30^ In cell-penetrating oligonucleotide phosphorothioates, the negatively charged sulfur atoms could behave like non-protonatable pseudo-thiolates^10,31–33^ and enter into cells by dynamic covalent cascade exchange with cellular disulfides and thiol/ates along the cellular redox gradient. This observation implied that lipid phosphorothioates could enable TMU of liposomes. This implication was interesting because liposomal drug delivery has attracted extensive scientific attention,^34–41^ including an early example^42^ and more recent progress^43–46^ to integrate TMU with maleimides^42,43^ and different disulfides.^44–49^ A recent example combines oligonucleotide phosphorothioates with liposomes for delivery and genome editing.^50^ In this report, we provide experimental support that liposomes made from phosphorothioate lipids can enter cells by TMU (Fig. 1d and e).

**Fig. 1:**
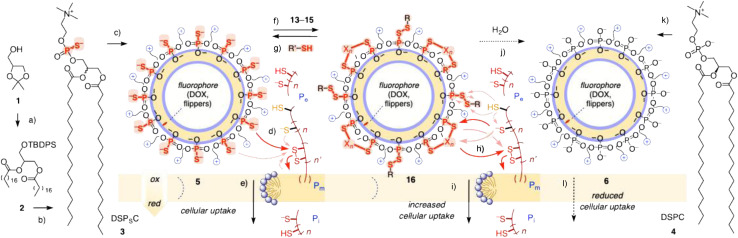
(a and b) Synthesis of phosphorothioate lipids DSP_S_C 3 and (c) their assembly into liposomes 5 labeled with DOX or flippers for (d and e) TMU through reorganized membrane domains like toroidal elastics along the cellular redox gradient, compared to (k and l) LUV control 6 from DSPC 4. (f and g) Exchange of LUVs 5 with 13–15 to yield surface-modified LUVs 16 for (h and i) enhanced TMU, and (j) their potential hydrolysis into LUVs 6. (a) 1. TBDPSCl, DMAP, Et_3_N, CH_2_Cl_2_, rt, 16 h, 93%; 2. HCl_aq_, MeOH, CH_2_Cl_2_, rt, 2 h, 60%; 3. Stearic acid, DCC, DMAP, CH_2_Cl_2_, rt, 16 h, 92%; 4. TBAF, AcOH, THF, 0 °C to rt, 77%.^51,52^ (b) 1. SPCl_3_, Et_3_N, CHCl_3_, 45 °C, 30 min; 2. Choline toluenesulfonate, pyridine, rt, 18 h; 3. H_2_O, rt, 30 min, 30%. (d and h) Possible exchange cascades with cellular thiols and disulfide on extracellular (*P*_e_), intracellular (*P*_i_) and exchange partners in the plasma membrane (*P*_m_). See Fig. 2 for the structures of 13–15.

Phosphorothioate lipids were synthesized from glycerol acetal 1 following the reported protocols for similar lipids (Fig. 1a, b and Scheme S1).^51–55^ Silyl protection of the primary alcohol followed by de-acetalization and esterification with fatty acids of free choice affords intermediate 2. The phosphorothioate is then installed with SPCl_3_ together with phosphocholine as the conventional headgroup (Fig. 1b). Along this route, phosphorothioates DSP_S_C 3 were prepared as a mixture of two diastereomers in racemic form. The corresponding native phosphodiester lipids DSPC 4 were commercially available in enantiopure form (*R*-distearoylphosphatidylcholine). Phosphorothioate large-unilamellar vesicles (LUVs) 5 and phosphodiester controls 6 were prepared by conventional freeze-thaw extrusion techniques. The expected uniform diameter (120 to 140 nm) and negligible *ζ*-potential (−0.8 to −5.7 mV) were confirmed by DLS (dynamic light scattering, Fig. S9). The compatibility of DSP_S_C LUVs with dynamic covalent phosphorothioate chemistry was confirmed by exchange with DTNB (5,5′-dithiobis-(2-nitrobenzoic acid)). The formation of phosphorothioate pseudo-disulfides on the vesicle surface was evinced by the absorption around 420 nm of the released push–pull thiophenolate (Fig. 2a and S10).

**Fig. 2:**
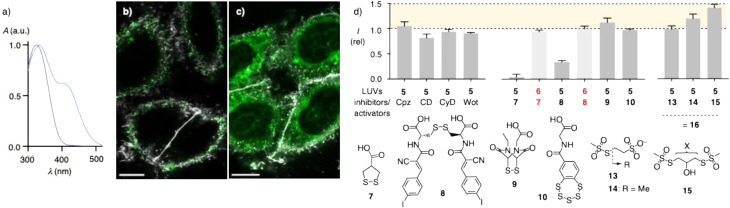
(a) Normalized absorption spectra of DTNB (1 eq/lipid) after addition to DSP_S_C LUVs 5 (blue) and DSPC LUVs 6 (purple, 5 mM HEPES, 172 mM NaCl, pH 7.4). (b and c) CLSM images of HK cells incubated for 2 h with DOX-loaded DSPC LUVs 6 (b) and DSP_S_C LUVs 5 (c, green), and a plasma membrane probe (grey, scale bars = 10 μm, laser power and brightness individually adjusted). (d) Relative fluorescent intensity of HK cells incubated with DSP_S_C LUVs 5 or DSPC LUVs 6 without (*I*_0_, *I*_rel_ = *I*_0_/*I*_0_ = 1) and with endocytosis inhibitors chlorpromazine (Cpz), mβCD (CD), cytochalasin D (CyD) and wortmannin (Wot), TMU inhibitors 7–10 and TMU activators 13–15 (*I*, *I*_rel_ = *I*/*I*_0_).

TMU is routinely assessed in HeLa Kyoto (HK) human cancer cells, similar results with meaningful variations are usually obtained in other cells.^5,11–30^ To follow cellular uptake by confocal laser scanning microscopy (CLSM), LUVs were loaded with doxorubicin (DOX), a DNA-intercalating antitumor natural product that is commonly used in studies on liposomal delivery (Fig. 1).^47,56–58^ To differentiate fluorescent liposomes inside cells from liposomes bound to the cell surface, the use of CLSM imaging with counter-staining of the plasma membrane (PM) was essential. DOX-loaded DSPC LUVs 6 (green) co-localized with PM trackers (grey), indicating that they did not penetrate HK cells under the selected conditions (Fig. 2b). In clear contrast, phosphorothioate DSP_S_C LUVs 5 produced diffuse staining, excluding nuclei, along with puncta within the area bordered by plasma membranes, indicating their uptake into the cytosol (Fig. 2c). The number of live HK cells was not affected by treatment with DOX-loaded LUVs, consistent with DOX localization in the cytosol rather than the nucleus. Mixed DSP_S_C/DSPC LUVs with 33% and 66% DSP_S_C showed a gradual transition from surface binding to cytosolic delivery (Fig. S24). Uptake and differences in uptake were visible already after 30 minutes, most distinct after two hours, and continued to increase until four hours of incubation (Fig. S21).

According to image-based high-throughput analysis, the entry of phosphorothioate DSP_S_C LUVs 5 was barely affected by common inhibitors of different types of endocytosis.^51,52,59–62^ Examples include chlorpromazine (Cpz) for clathrin-mediated and methyl-β-cyclodextrin (CD) for caveolar endocytosis, cytochalasin D (CyD) and wortmannin (Wot) for phagocytosis and macropinocytosis (Fig. 2d). Control experiments with fluorescent EGF and dextran confirmed that the inhibitors were active under the selected concentrations (Fig. S28).

Standard TMU inhibitors 7–10 were prepared following reported procedures^63–66^ and tested for their activities under standard pre-incubation conditions (Fig. 2d).^67^ Namely, HK cells were incubated with inhibitors and rinsed prior to the treatment with labeled LUVs. Cellular uptake of DSP_S_C LUVs 5 was efficiently inhibited by AspA 7 and the reversible Michael acceptors^66,68^8. Uptake inhibition by AspA 7 with an IC_50_ = 25 ± 4 μM was particularly interesting because this original cascade exchanger (CAX) is usually a comparably poor inhibitor, although not as poor as the often used DTNB.^67,69^ In contrast, the often more powerful^67^ inhibitors ETP 9 or BPS 10 were inactive. This selectivity pattern was as expected, given the existence of multiple almost orthogonal exchange networks in TMU.^67^ It supported the previous hypothesis^67^ that phosphorothioates engage in the exchange network accounting for TMU of AspA derivatives, which is likely to include the transferrin receptor,^70^ among other exchange partners,^71^ while the integrins and PDIs from the ETP and BPS pathways appear less compatible with TMU of phosphorothioates.^67^ Uptake of TMU-incompatible phosphodiester DSPC LUVs 6 was not inhibited, also by the best DSP_S_C inhibitors 7 and 8 (Fig. 2d).

To elaborate on the nature and cellular uptake of phosphorothioate liposomes, fluorescent flippers 11 or 12 were inserted into their membrane (Fig. 3a). Fluorescent flippers are bioinspired^72–74^ planarizable push–pull probes that have been introduced^75^ to image the order and changes in tension of biomembranes.^76–79^ Mechanical compression forces the two twisted dithienothiophene chromophores into conjugation and generates a push–pull system that red shifts excitation maxima and increases fluorescent intensity and lifetimes.

**Fig. 3:**
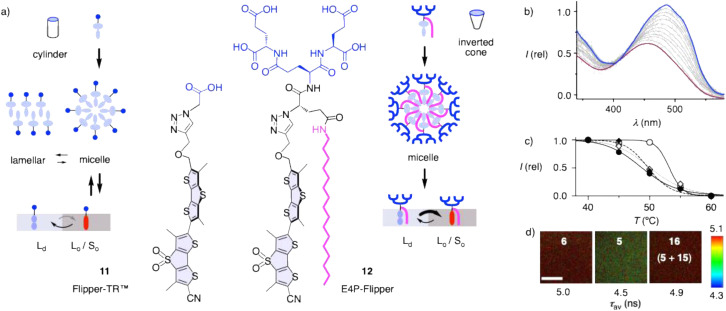
(a) The inverted-cone shaped palmitoylated flippers 12 form stable micelles in water and bind irreversibly to more ordered membranes (*L*_o_, *S*_o_), while the original cylinder-shaped flippers 11 form less stable micelles and reversibly bind to less ordered membranes (*L*_d_). (b) Excitation spectra of DSP_S_C LUVs 5 with 0.01% 11, decreasing with increasing temperature from 40 (blue) to 55 °C (red, *λ*_em_ = 650 nm). (c) Temperature dependence of normalized fluorescence intensity of 11 (0.01%) in DSPC LUVs 6 (empty circles), DSP_S_C LUVs 5 (filled circles), and DSP_S_C LUVs 16 surface-modified with 14 (filled diamonds) and 15 (empty diamonds). (d) FLIM images of 12 (1%) in DSPC LUVs 6, DSP_S_C LUVs 5 and DSP_S_C LUVs 16 surface-modified with 15, with fluorescent lifetimes (DOPC: *τ* = 3.1 ns, Fig. S29; scale bar = 100 μm).

In water, flipper fluorescence is quenched by self-assembly into micelles. In liquid-disordered (*L*_d_) membranes, the mechanical compression by lipids is insufficient to fully planarize flippers, which is reflected in blue-shifted excitation maxima and short fluorescence lifetime. Stronger planarization in liquid-ordered (*L*_o_) and solid-ordered (*S*_o_) membranes red shifts excitation maxima and increases lifetime. In phosphorothioate DSP_S_C LUVs 5, the original flipper probe 11 showed the typical temperature-dependent excitation spectra with intense bathochromic peaks at low temperatures and a weak hypsochromic band at high temperatures, consistent with *S*_o_ membranes melting into *L*_d_ membranes (Fig. 3b). Thus, this spectral change allows the determination of the phase transition temperature (*T*_M_). Compared to the sharp phase transition of enantiopure DSPC LUVs 6 at the known *T*_M_ = 55 °C,^80^ DSP_S_C LUVs 5 showed a more gradual transition at a lower *T*_M_ = 48 °C, possibly due to the presence of four different stereoisomers (Fig. 3c, filled circles).^81–83^

For bioimaging with flipper probes, fluorescence lifetime imaging microscopy (FLIM) is used to conveniently and reliably report on changes in membrane order and tension. Fluorescent lifetimes *τ* were determined by fit-free phasor analysis.^84^ To trace liposomal delivery, the original flipper 11 is not well suited because its partitioning in membranes is reversible (Fig. 3a).^85^ The flipper dendrimers 12 were introduced last year to achieve nearly irreversible membrane partitioning.^85^ As inverted cones, they afford essential^86^ stable micelles in water without competing precipitation into lamellar solids, which increases membrane labeling and, thus, effective brightness. Palmitoylation assures irreversible membrane binding with preference for more ordered membrane domains, while anionic dendrons will further suppress intrinsically disfavored^87^ transmembrane interleaflet transfer (flip-flop).

FLIM of flippers 12 in DSP_S_C LUVs 5 gave *τ* = 4.5 ns, consistent with *S*_o_ membranes (Fig. 3d). Higher order of enantiopure DSPC LUVs 6, reflected in the sharp *T*_M_ = 55 °C, was faithfully reported as a longer lifetime *τ* = 5.0 ns. In comparison, single-component *L*_d_ membranes of DOPC LUVs gave a much smaller *τ* = 3.1 ns (Fig. S29).

FLIM images of HK cells treated with phosphodiester DSPC LUVs 6 labeled with mechanosensitive LUV trackers 12 were similar in appearance to the CLSM images with DOX-loaded LUVs (Fig. 2b) and showed puncta on the plasma membrane with *τ* = 4.9 ns (Fig. 4c). These results suggested that phosphodiester DSPC LUVs 6 bind to the cell surface but neither fuse with the plasma membrane nor engage in significant endocytosis under the selected conditions for up to 4 hours.

**Fig. 4:**
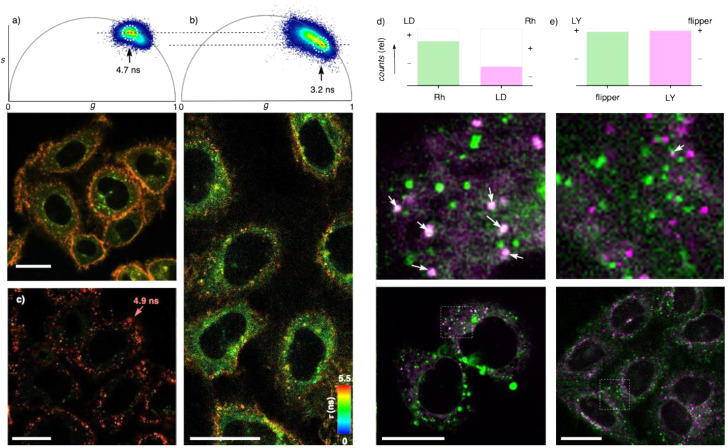
(a–c) FLIM images of HK cells incubated for 2 h with (a) flipper 12 and (b) DSP_S_C LUVs 5 and (c) DSPC LUVs 6, both with 1% 12, with phasor plots^84^ for fit-free lifetime analysis for (a) and (b, top). (d) CLSM image of HK cells incubated with DSP_S_C LUVs 5 containing 1% Rh-DPPE (green) and lipid droplet tracker (LD, magenta, bottom), with zoom (middle) and relative counts of LD tracker without (magenta) and with co-localization with Rh (white, top right), and Rh without (green) and with co-localization with LD tracker (white, top left). (e) CLSM image of HK cells incubated with DSP_S_C LUVs 5 containing 1% flipper 12 (green) and LysoTracker (LY, magenta, bottom), with zoom (middle) and relative counts of LY tracker without (magenta) and with co-localization with flipper (white) and flipper without (green) and with co-localization with LY tracker (white, top); scale bars = 20 μm.

In contrast, phosphorothioate DSP_S_C LUVs 5 labeled with flippers 12 added to HK cells produced signals mainly between the plasma membrane and nucleus, thus presumably in the cytoplasm (Fig. 4b). The short lifetime maximizing in the phasor plot at *τ* = 3.2 ns revealed that after uptake with the *S*_o_ phosphorothioate liposomes 5, reported at *τ* = 4.5 ns (Fig. 3d), the environment of flippers 12 becomes clearly more disordered (Fig. 4b). Similarly short lifetimes were previously reported with flipper probes targeted to organellar membranes, such as endoplasmic reticulum.^87,88^ The less important longer lifetimes observable in FLIM images and phasor plots were localized around the plasma membrane (Fig. 4b).

Flippers 12 added directly to the cells under otherwise identical conditions mostly labeled the highly ordered plasma membrane with a *τ* = 4.7 ns (Fig. 4a). The apparent difference between FLIM images of flippers 12 added without and with DSP_S_C LUVs 5 to HK cells was qualitatively confirmed in phasor plots (Fig. 4a *vs.* b). These results indicated that the observed uptake does not arise from the thiol-mediated fusion of DSP_S_C LUVs 5 with the plasma membrane, leading to the release of flippers in the plasma membrane, which are taken up through conventional biological mechanisms, primarily endocytosis. The difference between these FLIM images thus supported that DSP_S_C LUVs 5 remain intact while crossing the plasma membrane by TMU along the cellular redox gradient, presumably through toroidal elastics or related membrane deformations (Fig. 1, previous studies reported decreasing lifetimes of flippers in plasma membranes during TMU, consistent with local membrane disorganization and/or decreasing membrane tension^5,69^).

Little release of DOX into the nucleus (Fig. 2c) supported that DSP_S_C LUVs 5 remain at least partially intact after uptake. Considering slow intermembrane transfer of dendron 12,^85^ decreasing lifetimes from *τ* = 4.7 ns to *τ* = 3.2 ns of apparently intact liposomes suggested that the order of their membrane and thus their lipid composition changes in the cytosol. Fusion with lysosomes was unlikely because, with *τ* ∼ 3.9 ns,^76^ their membranes should be more ordered. This conclusion was validated by poor co-localization with LysoTracker™ (Fig. 4e, magenta). It provided corroborative support that DSP_S_C LUVs 5 do not enter cells by endocytosis, as indicated by insensitivity to endocytosis inhibitors (Fig. 2d).

The spectroscopic properties of available trackers of lipid droplets (LDs) were incompatible with co-localization experiments with flipper 12. DSP_S_C LUVs 5 were thus equipped with sulforhodamine B (Rh) attached to the DPPE amine for mechanoinsensitive LUV tracking. After TMU, many LDs co-localized with LUV trackers (Fig. 4d, white *vs.* magenta), while only a few LUV-positive puncta co-localized with LDs (Fig. 4d, white *vs.* green). This indicated that after TMU, DSP_S_C LUVs 5 are in contact with lipid droplets, but not exclusively. Such contacts appeared meaningful considering that lipid droplets are the site of lipid storage and metabolism,^89–91^ and perhaps useful to exchange lipids. Whereas the nature of LD-negative puncta is essentially unknown, their distinct shape, short fluorescence lifetime and unreleased content would be consistent with intact DSP_S_C LUVs 5 after the incorporation of disorganizing lipid components from lipid droplets.

Phosphorothioates can initiate dynamic covalent cascade exchange for TMU with cellular disulfides but not with cellular thiols (Fig. 1d).^5,11–16,18–27^ The possibility of activating them as pseudo-disulfides for exchanging with cellular thiols to initiate TMU raises intriguing questions about the poorly explored dynamic covalent chemistry of phosphorothioates.^10,32^ Consistent with earlier results,^10^ exchange of adenosine-5′-O-monophosphorothioate with thiosulfonates 13–15 was instantaneous in neutral buffer to afford pseudo-disulfides PSSR, which in turn exchanged with Ac-Cys-NH_2_ as a minimalist protein mimic (Fig. 1f, g and S2–S5). Competing inactivation by hydrolysis to phosphate esters was not observed under experimental conditions (Fig. 1j and S6; pure PSSP disulfides^33^ could not be realized in the context of phosphorothioate liposomes).

With dynamic covalent exchange on the surface of DSP_S_C LUVs 5 being detectable by DTNB (Fig. 2a), almost complete conversion of phosphorothioates to pseudo-disulfides on the liposome surface with thiosulfonates 13–15 was demonstrated by inhibition of this visible exchange with DTNB (Fig. 1f and S11). Melting curves recorded with flippers 11 demonstrated a slightly higher *T*_M_ of DSP_S_C LUVs 5 by treatment with doubly reactive 15, which would be consistent with the formation of organizing, cardiolipin-like lipid dimers in DSP_S_C LUVs 16. The *T*_M_ recorded for DSP_S_C LUVs 5 with 14 was similar, but the melting curve was steeper, implying the formation of cationic RSSP lipid monomers (Fig. 3c and 1f). More important changes could not be expected considering the poor sensitivity of *T*_M_ even to massive headgroup modifications in biological membranes (*e.g.*, *T*_M_ of neutral PC and anionic PG are the same). Increasing membrane order with cardiolipin-like lipid dimers was reported in FLIM images of flippers 12 (Fig. 3d). Fluorescence lifetimes increased from *τ* = 4.5 ns to *τ* = 4.9 ns for possibly crosslinked DSP_S_C LUVs 16, a value near the *τ* = 5.0 ns of the enantiopure phosphodiester DSPC LUVs 6.

TMU of DSP_S_C LUVs 5 increased slightly upon pre-activation with thiosulfonates 14 and particularly 15, but not with 13 (Fig. 2d). These trends were consistent with increasing positive charge and dimerizing pseudo-disulfides on the surface of DSP_S_C LUVs 16. However, the changes were nearly negligible, suggesting that pseudo-disulfides cleaved easily (Fig. 1g), or partial hydrolysis into inactive DSPC LUVs 6 concealed more important activation (Fig. 1j). Overall, activation of DSP_S_C LUVs 5 as DSP_S_C LUVs 16 with pseudo-disulfides on their surface gave meaningful trends for all aspects covered in this study, but the observed changes were very small, most notable for increasing membrane order in LUVs reported by flipper probes (Fig. 3d).

In summary, by translating lessons from oligonucleotides, we show that the replacement of a single oxygen by a sulfur atom in biological phospholipids can afford cell-penetrating phosphorothioate liposomes. Dynamic covalent exchange cascades of phosphorothioates with cellular thiols and disulfides, that is thiol-mediated uptake, are shown to account for the delivery of liposomes without endocytosis and little fusion at the plasma membrane. In the cytosol, the liposomes do not release their contents, while their membranes become highly disorganized, presumably by integrating disorganizing lipids from lipid droplets. Valuable mechanistic insights are obtained with new flipper dendrons, which thus emerge as useful fluorescent tools, mechanosensitive LUV trackers, to elucidate liposomal delivery in general.

## Experimental section

See SI.

## Author contributions

J. B. and F. B. performed all synthesis, J. B. and N. S. liposome characterization and cellular uptake studies, N. S. and S. M. directed the study, all authors contributed to the design of experiments, data interpretation and manuscript writing.

## Conflicts of interest

The authors declare the following competing financial interest: The University of Geneva has licensed four Flipper-TR^®^ probes to Spirochrome for commercialization.

## Supplementary Material

SC-016-D5SC05796E-s001

## Data Availability

Data for this paper are available at Zenodo at https://doi.org/10.5281/zenodo.17043219. Supplementary information: detailed procedures and results for all reported experiments. See DOI: https://doi.org/10.1039/d5sc05796e.
