# Supplementary material for: Thiol-mediated uptake of phosphorothioate liposomes, visualized with fluorescent flippers
Source: Chem Sci. 2025 Sep 29;16(40):18599–606. doi: 10.1039/d5sc05796e (PMC12478204; doi:10.1039/d5sc05796e)
Supplement: SC-016-D5SC05796E-s001 [file SC-016-D5SC05796E-s001.pdf]

Supplementary Information (SI) for Chemical Science.
This journal is © The Royal Society of Chemistry 2025

Electronic supplementary information

Thiol-mediated uptake of phosphorothioate liposomes,

visualized with fluorescent flippers

Jules Bouffard, Felix Bayard, Naomi Sakai and Stefan Matile*

Department of Organic Chemistry, University of Geneva, CH-1211 Geneva, Switzerland

*E-mail: stefan.matile@unige.ch

Table of contents

1. Materials and methods S4

2. Synthesis S6

   2.1. DSP_SC synthesis S6

   2.2. Thiosulfonate synthesis S7

   2.3. Synthesis of inhibitors and flippers S8

3. Phosphorothioate/thiosulfonate exchange studies S9

   3.1. Thiosulfonate exchange with 5'-AMPS S9

   3.2. Disulfide 5'-AMPS exchange with cysteine S11

   3.3. Thiosulfonate exchange with *O,O*-diethylthiophosphate S12

4. LUVs formation S14

   4.1. Preparation of empty LUVs S14

   4.2. Doxorubicin encapsulated LUVs S14

      4.2.1. Detection of encapsulated doxorubicin S15

      4.2.2. LUVs ⊃ DOX purification S16

   4.3. E4P-Flipper LUVs preparation for cellular uptake S17

5. LUVs characterization S17

   5.1. Dynamic light scattering S17

   5.2. Surface modification quantification by DTNB assay S18

   5.3. Flipper properties in LUVs S21

      5.3.1. Concentration dependence S21

      5.3.2. Temperature dependence S22

      5.3.3. Extraction of **11** or **12** with BSA from LUVs S23

6. Cell culture S25

7. Evaluation of LUVs uptake by CLSM S25

7.1. General experimental procedure S25

7.2. Data analysis S26

7.3. Results for uptake into HK cells S28

7.3.1. LUVs ⊃ DOX S28

7.3.2. LUVs ⊃ E4P-Flipper S34

8. AHCHT uptake inhibition assays S35

8.1. General experimental procedure S35

8.2. Data analysis S37

8.3. Results S38

8.3.1. With TMU inhibitors S38

8.3.2. With endocytosis inhibitors S41

9. Evaluation of LUVs uptake by FLIM S42

9.1. Fluorescence lifetime determination by FLIM imaging of LUVs S42

9.2. Imaging uptake of LUVs in HK cells by FLIM S42

10. Co-localization S44

11. Supplementary references S46

12. NMR spectra S48

1. Materials and methods

As in reference S1. Briefly, reagents for synthesis were purchased from Fluka, Sigma-Aldrich, TCI, and Across. Salts of the best grade available from Fluka or Sigma-Aldrich were used as received. 1,2-dioleoyl-*sn*-glycero-3-phosphocholine (DOPC), 1,2-distearoyl-*sn*-glycero-3-phosphocholine (DSPC), 1,2-dipalmitoyl-sn-glycero-3-phosphoethanolamine-N-(lissamine rhodamine B sulfonyl) (16:0 Liss Rhod PE), and Mini-extruder were purchased from Avanti Polar Lipids. Dulbecco's phosphate buffered saline (DPBS, no Ca^{2+}, no Mg^{2+}, pH = 7.4), Leibovitz's L-15 medium, FluoroBrite DMEM (high D-Glucose) medium, DMEM (high D-Glucose, with phenol red) with GlutaMAX supplement, Penicillin-Streptomycin, Fetal Bovine Serum, TrypLE Express Enzyme, and LysoTracker™ Red DND-99 were obtained from Thermo Fisher Scientific. BODIPY 493/503 was obtained from Cayman. 35 mm glass-bottom dishes were obtained from MatTek (P35G-0.170 14-C). 96-well μ-plates and μ-Slide 18-Well Glass Bottom were obtained from Ibidi. Flash column chromatography was performed on a Biotage Isolera™ system. Analytical (TLC) thin layer chromatography was performed on silica gel 60 F254.

Fluorescence cellular imaging was performed using Leica SP8 confocal equipped with a 63X oil immersion objective lens, or an IXM-C automated microscope from ImageXpress equipped with a Lumencor Aura III with solid-state light sources, bandpass filters, and a 10X objective. Sample preparation and washing on 96-well plates was performed using a Plate washer Biotek EL406®. Fluorescence lifetime imaging microscopy (FLIM) images were obtained using Leica Stellaris 8 Falcon or Leica SP8DIVE Falcon using a 63X oil immersion objective lens and analyzed with Leica LAS X FLIM/FCS software (4.5.0).

IR spectra were recorded on a Perkin Elmer Spectrum Two™ FT-IR spectrometer (ATR, Golden Gate) and are reported as wavenumbers υ in cm^{-1} with band intensities indicated as s (strong), m (medium), w (weak), br (broad). Dynamic light scattering (DLS) and zeta potential were recorded on a Malvern Zetasizer Nano ZS instrument using a folded capillary zeta cell. Fluorescence spectra

were recorded using a Horiba FluoroMax-4 spectrofluorometer equipped with a stirrer and a temperature controller. Fluorescence spectra were corrected for lamp intensity fluctuations, background, and the wavelength-dependent response function of the detector. UV-vis absorption measurements were performed on a JASCO V-650 spectrometer equipped with a temperature controller. All ^{1}H, ^{13}C, and ^{31}P NMR spectra were recorded (as indicated) on a Bruker 300 or 400 MHz spectrometer at room temperature (25 °C) and are reported as chemical shifts (δ) in ppm relative to TMS (δ = 0). Spin multiplicities are reported as singlet (s), doublet (d), triplet (t), and quartet (q) with coupling constants (*J*) given in Hz, or multiplet (m). Broad peaks are marked as br. ^{1}H and ^{13}C resonances were assigned with the aid of additional information from 1D and 2D NMR spectra (H, H-NOESY, H, H-COSY, DEPT 135, HSQC, and HMBC). ESI-HRMS was measured on Xevo G2-S Tof (Waters). All mass data are reported as mass-per-charge ratio *m/z*.

Abbreviations. 16:0 Liss Rhod PE: 1,2-Dipalmitoyl-sn-glycero-3-phosphoethanolamine-N-(lissamine rhodamine B sulfonyl); AHCHT: Automated high-content high-throughput; AspA: Asparagusic acid; BSA: Bovine serum albumin; Calcd.: Calculated; CAX: Covalent exchangers; CD: Methyl-β-cyclodextrin; CLSM: Confocal laser scanning microscopy; Cpz: chlorpromazine; CyD: cytochalasin D; DCC: N,N'-Dicyclohexylcarbodiimide; DLS: Dynamic Light Scattering; DMAP: 4-(Dimethylamino)pyridine; DMEM: Dulbecco's modified Eagle medium; DOX: Doxorubicin; DPBS: Dulbecco's phosphate-buffered saline; DSPC: 1,2-Distearoyl-sn-glycero-3-phosphocholine; DSP_SC: 1,2-Distearoyl-glycero-3-thiophosphocholine; DTNB: 5,5′-dithiobis-(2-nitrobenzoic acid): dMAC: Double Michael acceptor; EGF: Epidermal growth factor; FBS: Fetal bovine serum; FDMEM: FluoroBrite DMEM; FLIM: Fluorescence lifetime imaging microscopy; HEPES: 4-(2-Hydroxyethyl)piperazine-1-ethanesulfonic acid; HK: HeLa Kyoto; HRMS: High-resolution mass spectroscopy; IC50: Half maximal inhibitory concentration; L-15: Leibovitz's L-15 medium; LUV: Large unilamellar vesicles; MAC: Michael acceptor; MIC: Minimum inhibitory concentration; PBS: Phosphate-buffered saline; PM: Plasma membrane; ROI: Region of interest; rt: Room temperature;

RV: Relative viability; SD: Standard deviation; SDCM: Spinning disk confocal microscopy; SEM: Standard error of the mean; TBAF: Tetrabutylammonium fluoride; TBDPSCl: tert-Butyl(chloro)diphenylsilane; THF: tetrahydrofuran; Wot: wortmannin.

2. Synthesis

2.1. DSPsC synthesis

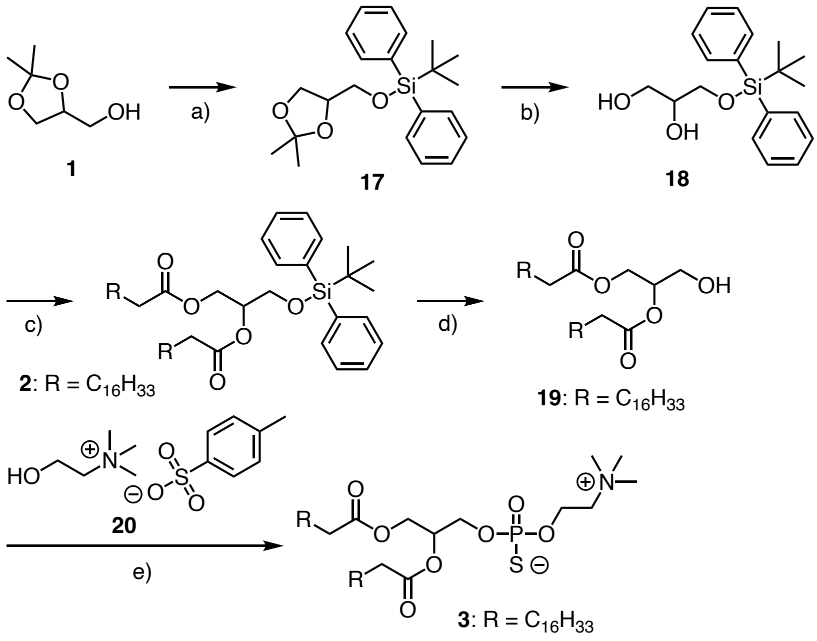

Scheme S1 Synthesis of substrates **3**. (a) TBDPSCl, DMAP, Et_3N, CH_2Cl_2, rt, 16 h, 93%. (b) HCl_{aq}, MeOH, CH_2Cl_2, rt, 2 h, 60%. (c) DCC, DMAP, stearic acid, CH_2Cl_2, rt, 16 h, 92%. (d) TBAF, AcOH, THF, 0 °C to rt, 77%. (e) 1. $SPCl_3$, Et_3N, $CHCl_3$, 45 °C, 30 min; 2. pyridine, choline toluenesulfonate **20**, rt, 18 h; 3. H_2O, rt, 30 min, 30%.

Compounds 17 and 18 were prepared following the reporting procedure[S2] starting with the racemic mixture of **1**. The experimental properties were consistent with those described in reference S2.

Compounds 2 and 19 were prepared following the reported procedure.[S3] The experimental properties were consistent with those described in reference S3.

Compound 20 was prepared following the reported procedure.[S4] The experimental properties were consistent with those described in reference S4.

Compound 3 was prepared by modifying the reported procedure.[S4] To $PSCl_3$ (100 µL, 1.0 mmol) under argon atmosphere at rt was added a solution of compound **19** (500 mg, 0.80 mmol) and dry Et_3N (140 µL, 1.0 mmol) in $CHCl_3$ (6 mL) dropwise within 5 min. The mixture was then heated at 45 °C for 30 min. After cooling to rt, dry pyridine (570 µL, 7.0 mmol) and **20** (370 mg, 1.4 mmol) were added and stirred for 18 h. Water (170 µL, 10 mmol) was then added and stirred for 30 min. The mixture was diluted with $CHCl_3$ (20 mL) and washed with 3% aqueous Na_2CO_3 (2 x 10 mL), 5% aqueous HCl (2 x 10 mL), and water (2 x 10 mL). The organic phase was dried over anhydrous Na_2SO_4 and concentrated *in vacuo*. The crude product was purified by flash column chromatography ($CHCl_3$/MeOH/65% NH_4OH in H_2O, 80:18:2) to afford **3** (194 mg, 30%) as a colorless solid. R_f ($CHCl_3$/MeOH/65% NH_4OH in H_2O, 68:28:4): 0.58; IR (neat): 2916 (s, CH), 2849 (s, CH), 1736 (m, C=O), 1163 (s, C-C(=O)-O), 1097 (m, P-O-C), 1049 (m, C-O-C), 965 (N-C); ^{1}H NMR (400 MHz, $CDCl_3$): 5.29-5.17 (m, 1H), 4.53-4.33 (m, 3H), 4.19-4.00 (m, 3H), 3.98-3.90 (m, 1H), 3.86-3.79 (m, 1H), 3.40 (s, 9H), 2.33-2.25 (m, 4H), 1.62-1.53 (m, 4H), 1.25 (br s, 56H), 0.88 (t, $^3J_{H\text{-}H}$ = 7.4 Hz, 6H); ^{13}C NMR (101 MHz, $CDCl_3$): 173.8 (C=O), 173.4 (C=O), 70.5 (CH), 66.4 (CH_2), 64.1 (CH_2), 63.1 (CH_2), 59.7 (CH_2), 55.0 ($3CH_3$), 34.5 (CH_2), 34.3 (CH_2), 32.1 ($2CH_2$), 29.9 ($16CH_2$), 29.7 ($2CH_2$), 29.5 ($4CH_2$), 29.3 ($2CH_2$), 25.1 ($2CH_2$), 22.8 ($2CH_2$), 14.3 ($2CH_3$); ^{31}P NMR (162 MHz, $CDCl_3$): 55.9, 55.8; HRMS (ESI) calcd. for $C_{44}H_{88}NO_7PS$ ($[M+H]^+$): 806.6092, found: 806.6084.

2.2. Thiosulfonate synthesis

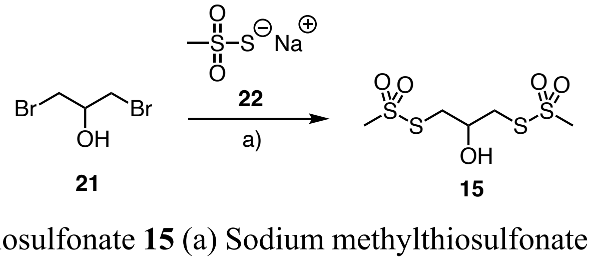

Scheme S2 Synthesis of thiosulfonate **15** (a) Sodium methylthiosulfonate **22**, DMF, 70 °C, 4 h, 95%.

Compound 15. A mixture of sodium methanethiosulfonate **22** (200 mg, 1.5 mmol) and **21** (160 mg, 0.70 mmol) in DMF (1.4 mL) was stirred for 4 h at 70 °C. Afterward, the solvent was evaporated *in vacuo,* and the residue was re-dissolved in CH_2Cl_2, washed with distilled water (2 x 10 mL), dried over anhydrous Na_2SO_4, and concentrated *in vacuo*. The crude product was purified by flash column chromatography (pentane/EtOAc, 50:50 to 0:100), to afford **15** as a colorless wax (190 mg, 95%). R_f (pentane/EtOAc, 1:1): 0.16; IR (neat): 3470 (m, OH), 3025 (w, CH), 1293 (s, SO), 1118 (s, SO), 1069 (s, C-O), 747 (s, C-S); ^{1}H NMR (400 MHz, $CDCl_3$): 4.37-4.29 (m, 1H), 3.47-3.41 (m, 8H), 3.34 (dd, $^{3}J_{H\text{-}H}$ = 14.8, 7.1 Hz, 2H), 2.91 (d, $^{3}J_{H\text{-}H}$ = 4.7 Hz, 1H); ^{13}C NMR (101 MHz, $CDCl_3$): 69.9 (CH), 50.8 ($2CH_3$), 41.6 ($2CH_2$).

2.3. Synthesis of inhibitors and flippers

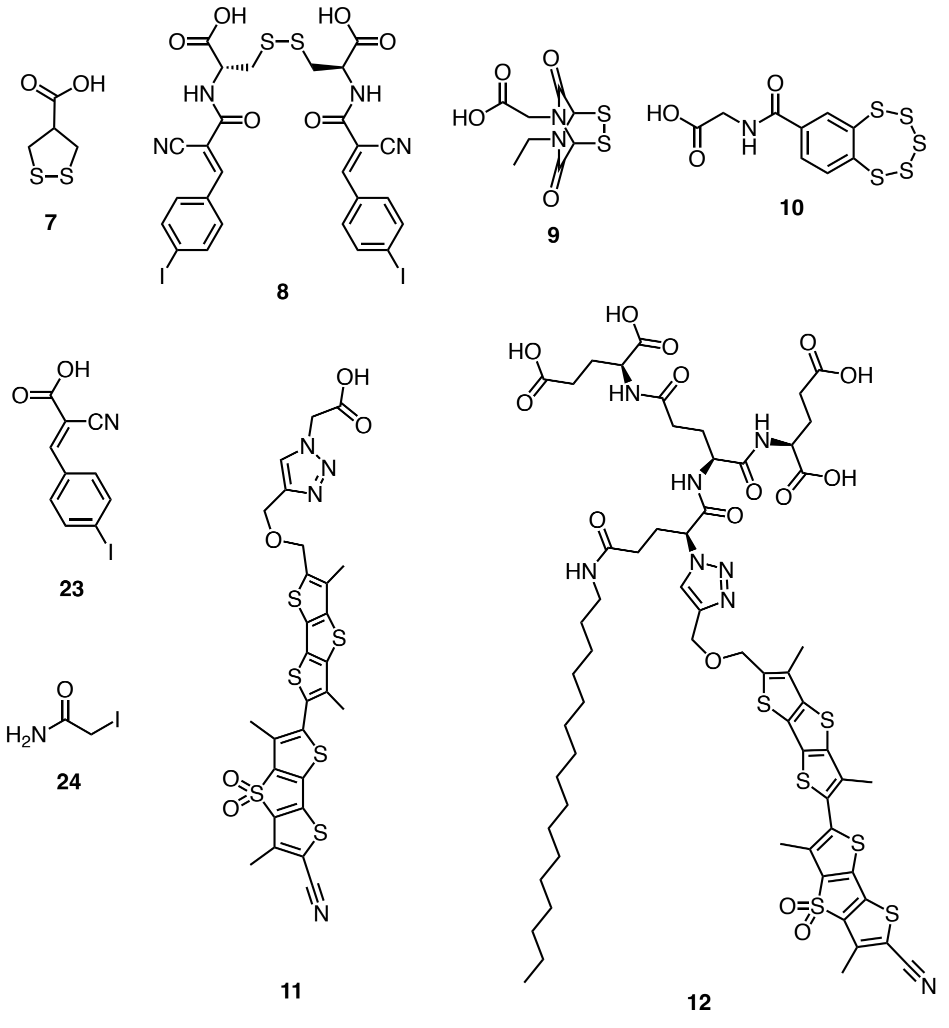

Fig. S1 Structures of CAX inhibitors (AspA **7**, dMAC **8**, ETP **9**, BPS **10**, MAC **23**, and iodoacetamide **24**) and flippers (Flipper TR® **11**, E4P-Flipper **12**).

AspA 7 was synthesized according to procedure described in reference S5.

ETP 9 was synthesized according to procedure described in reference S6.

BPS 10 was synthesized according to procedures described in reference S7.

dMAC 8, and **MAC 23** were synthesized according to procedures described in reference S8.

Flipper-TR® 11, and **E4P-Flipper 12** were synthesized according to procedures described in reference S9.

3. Phosphorothioate/thiosulfonate exchange studies

3.1. Thiosulfonate exchange with 5'-AMPS

UPLC-MS analyses were performed using Advion Avant® UHPLC system, with Advion Expression® CMS in ESI mode, with a column Hypersil Gold Vanquish 1.9 µm 2.1 x 50 mm, gradient: 5-95% CH_3CN in H_2O + 0.1% Formic acid in 4 min, 0.5 mL/min. UV (λ_{abs} = 260 nm) detection was used.

General procedure. As described in ref. S10. To a solution of 5'-AMPS **25** in PBS (1.0 mM, 4.0 µL, 1.0 eq.) was added a solution of thiosulfonate **13-15** in PBS buffer (0.1-10 eq, 36 µL, pH 7.4). After shaking for 30 min at rt, the mixture was analyzed by UPLC. Peak identities were verified by MS.

Compound 26 was prepared according to the general procedure using thiosulfonate **13** (11.1 µM (0.1 eq.), 55.5 µM (0.5 eq.), 111 µM (1 eq.), 555 µM (5 eq.) or 1.11 mM (10 eq.)).

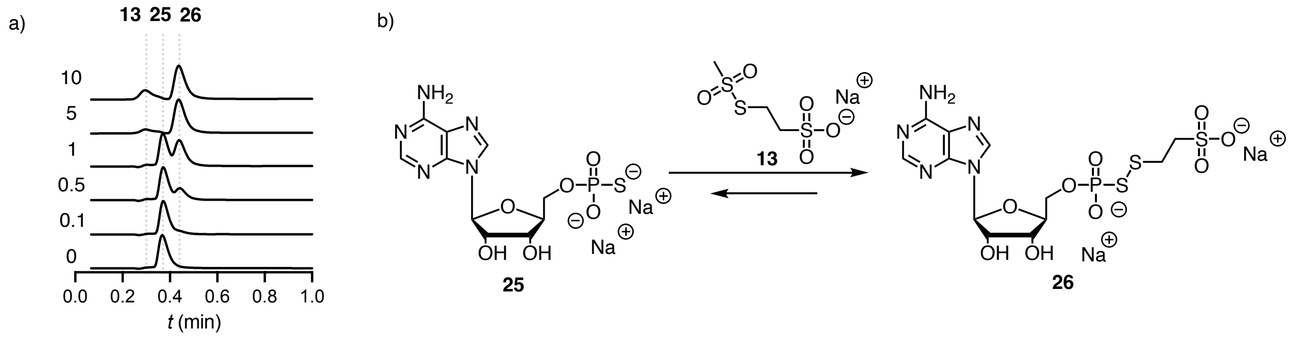

Fig. S2 (a) Normalized UPLC (λ_{abs} = 260 nm) chromatograms of **25** with 0, 0.1, 0.5, 1, 5, or 10 equiv. of **13** (bottom to top). (b) Reaction of 5'-AMPS **25** (t_R = 0.30 min) with thiosulfonate **13** (t_R = 0.37 min) to give **26** (t_R = 0.44 min).

Compound 27 was prepared according to the general procedure using thiosulfonate **14** (11.1 μM (0.1 eq.), 55.5 μM (0.5 eq.), 111 μM (1 eq.), 555 μM (5 eq.) or 1.11 mM (10 eq.)).

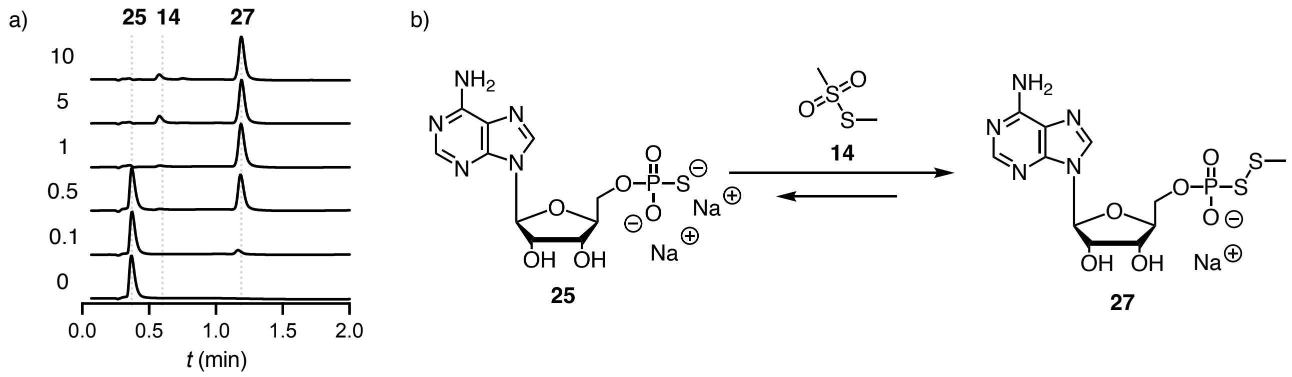

Fig. S3 (a) Normalized UPLC (λ_{abs} = 260 nm) chromatograms of **25** with 0, 0.1, 0.5, 1, 5, or 10 equiv. of **14** (bottom to top). (b) Reaction of 5'-AMPS **25** (t_R = 0.37 min) with thiosulfonate **14** (t_R = 0.6 min) to give **27** (t_R = 1.20 min).

Compounds 28-29 were prepared according to the general procedure using thiosulfonate **15** (11.1 μM (0.1 eq.), 111 μM (1 eq.), 222 μM (2 eq.), 555 μM (5 eq.) or 1.11 mM (10 eq.)).

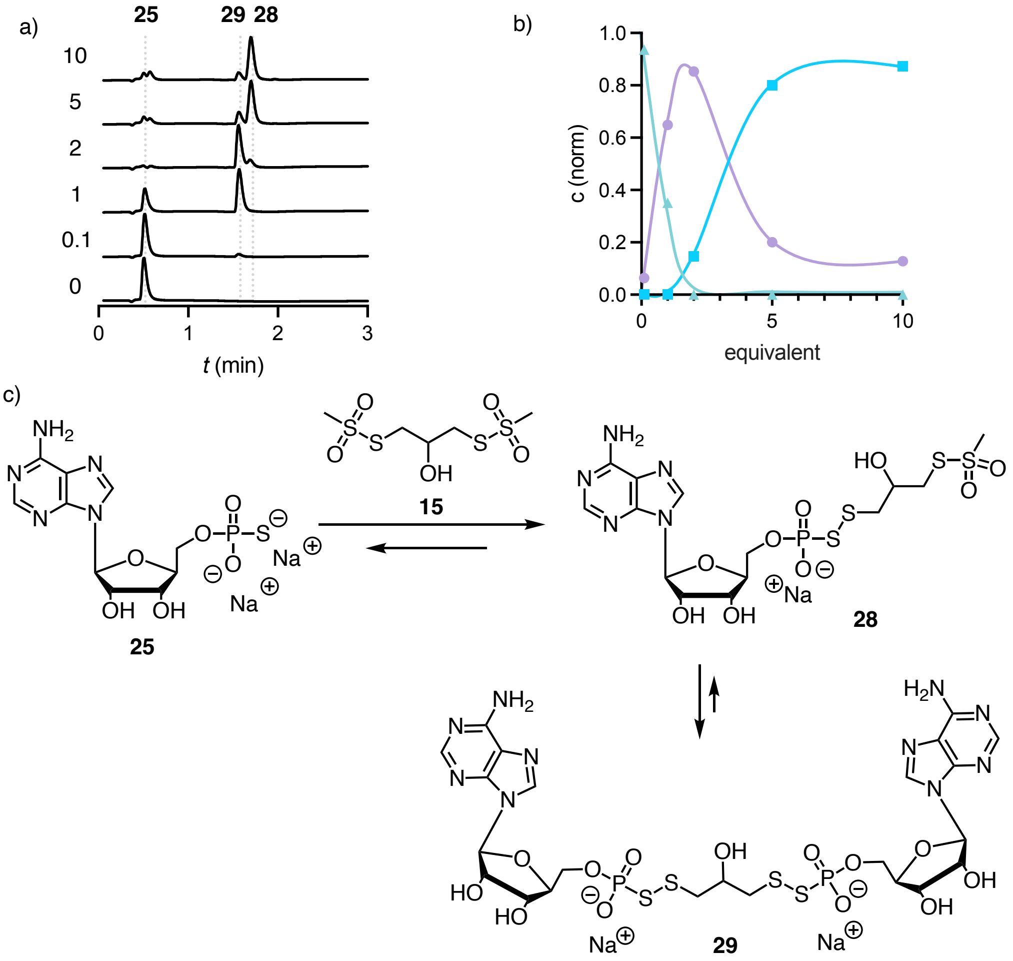

Fig. S4 (a) Normalized UPLC (λ_{abs} = 260 nm) chromatograms of **25** with 0, 0.1, 1, 2, 5, or 10 equiv. of **15** (bottom to top). (b) Exchange of 5'-AMPS **25** with different equivalents of **15** after 30 min (▲ **25**, ■ **28**, ● **29**). (c) Reaction of 5'-AMPS **25** with thiosulfonate **15** (t_R = 0.52 min) to give **29** (t_R = 1.58 min) or **28** (t_R = 1.72 min).

3.2. Disulfide 5'-AMPS exchange with cysteine

General procedure. To a solution of preactivated 5'-AMPS disulfide **26** or **27** in PBS (1 mM, 40 µL, 1.0 eq) was added a solution of cysteine derivative **30** in PBS buffer (10 µM (0.1 eq.), 20 µM (0.2 eq.), 50 µM (0.5 eq.), 100 µM (1 eq.), 400 µM (4 eq.) or 1.0 mM (10 eq.), 40 µL, pH 7.4). LC-MS chromatograms were measured after 30 min.

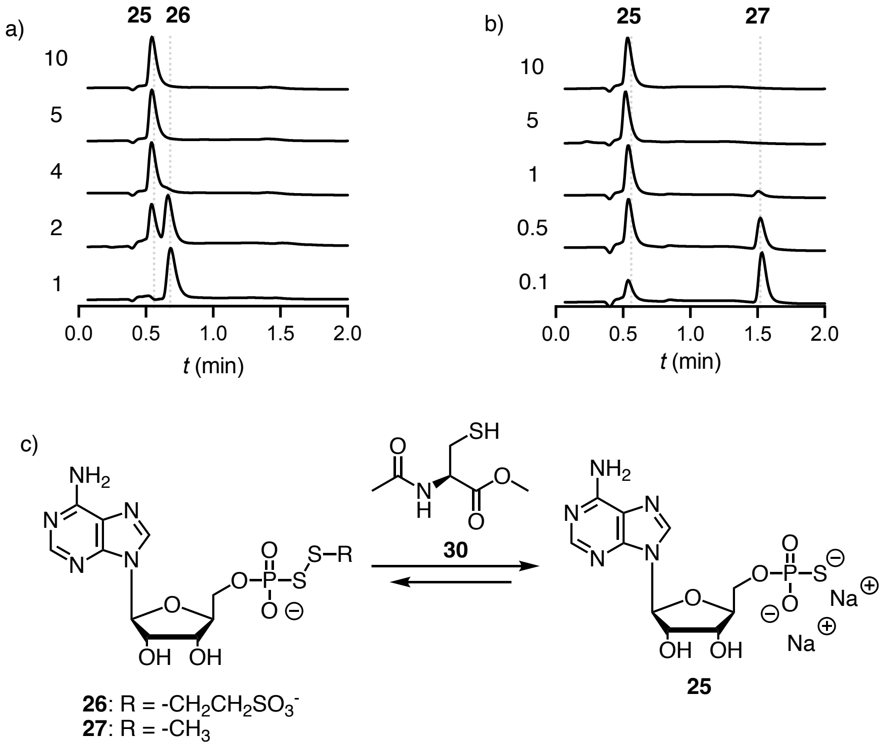

Fig. S5 (a) Normalized UPLC (λ_{abs} = 260 nm) chromatograms of **26** with 1, 2, 4, 5, or 10 equiv. of **30** (bottom to top). (b) Normalized UPLC (λ_{abs} = 260 nm) chromatograms of **27** with 0.1, 0.5, 1, 5, or 10 equiv. of **30** (bottom to top). (c) Reaction of **26** or **27** with cysteine **30**.

3.3. Thiosulfonate exchange with *O*,*O*-diethylthiophosphate

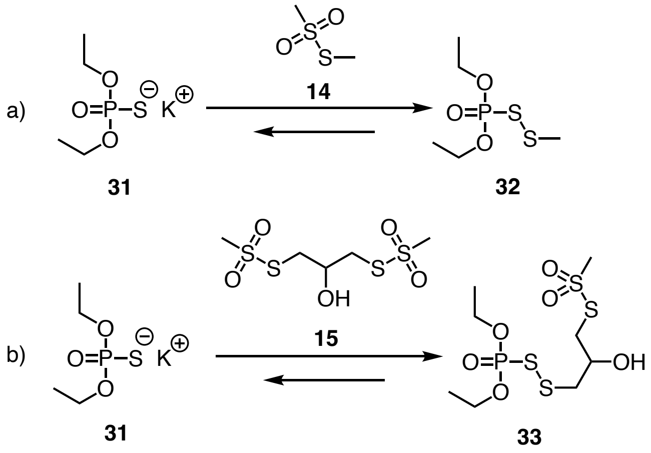

Scheme S3 Reaction of phosphorothioate **31** with **14** (a) or **15** (b).

General procedure. To a solution of **31** (100 µL, 50 mM in ethanol) was added a solution of thiosulfonate **14** or **15** (400 µL, 100 mM in ethanol) and stirred for 30 min. Afterward, CD_3OD (100 µL) was added to the solution and measured by quantitative ^{31}P NMR (121 MHz).

A control experiment was conducted by adding 100 µL of HEPES buffer (5 mM HEPES, 172 mM NaCl, pH 7.4) to the solutions of **31** and **14**, followed by stirring for 10 min before measurement by quantitative ^{31}P NMR (121 MHz).

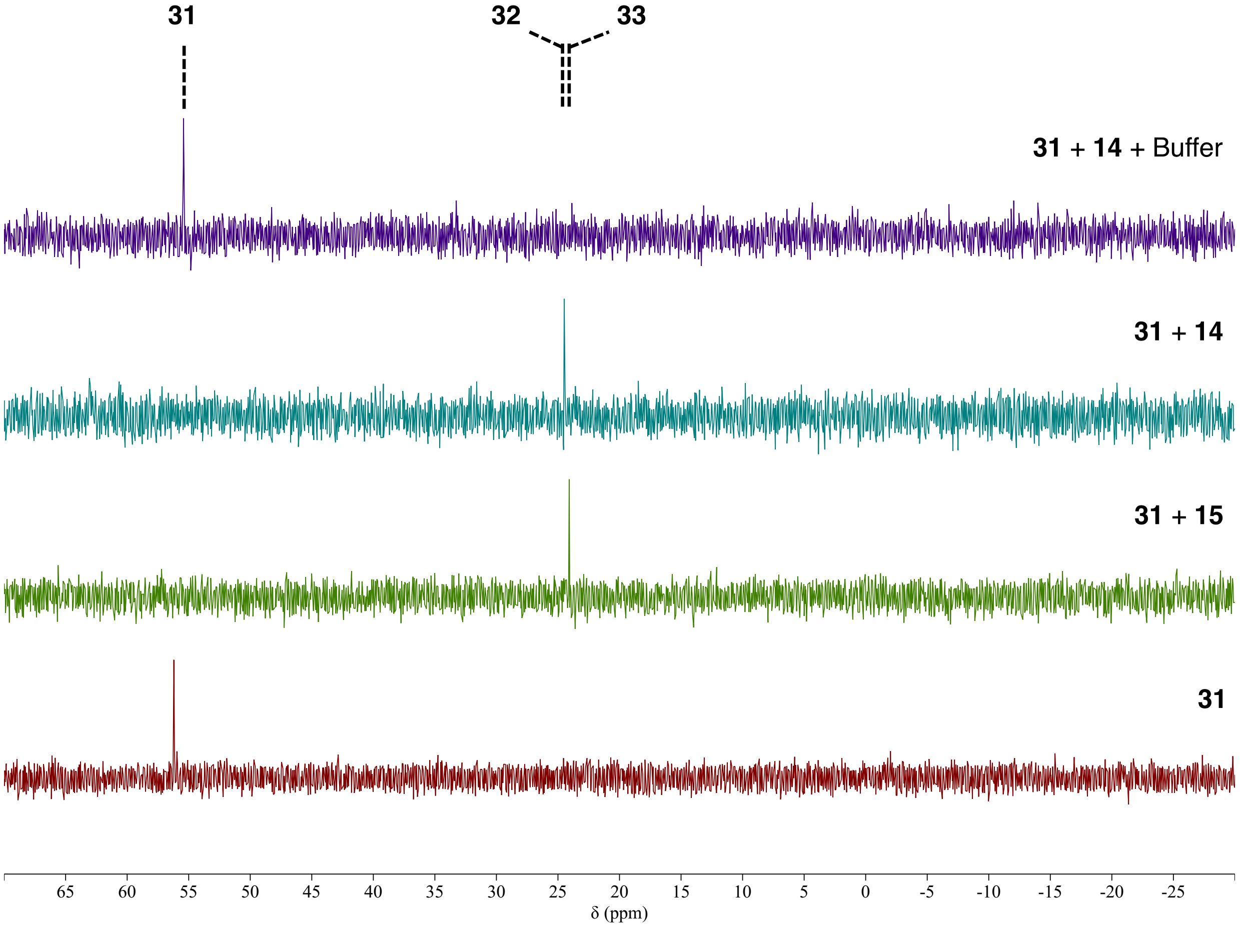

Fig. S6 Representative ^{31}P NMR (bottom to top) of phosphorothioate **31** alone (56.0 ppm), after addition of **15** (24.0 ppm), or **14** (24.5 ppm), and addition of **14** and HEPES buffer (55.5 ppm).

4. LUVs formation

4.1. Preparation of empty LUVs

DSP$_S$C LUVs ***5***. As described in reference S11. A thin lipid film was obtained by evaporating a solution of DSP$_S$C **3** (8 mg, 10 μmol) in MeOH/$CHCl_3$ 1:1 (1 mL) on a rotary evaporator and then drying it *in vacuo* overnight. The resulting film was hydrated with a buffer (1.0 mL, 5 mM HEPES, 172 mM NaCl, pH 7.4) at 65 °C for 60 min, subjected to freeze (liquid N_2)-thaw (65 °C) cycles (10x) and extrusions (15x, 65 °C) through a polycarbonate membrane (pore size: 100 nm). Final conditions: ~10 mM lipids; inside and outside: 5 mM HEPES, 172 mM NaCl, pH 7.4.

DSPC LUVs ***6*** were prepared similarly using DSPC **4** (8 mg, 10 μmol).

DSP$_S$C: 16:0 Liss Rhod PE LUVs ***35*** were prepared similarly using DSP$_S$C **3** (8.0 mg, 9.9 μmol) and 16:0 Liss Rhod PE **34** (20 μL of a 4.4 mM solution in $CHCl_3$, 0.1 μmol). Hydration and extrusion were performed at 65 ºC.

DSPC: 16:0 Liss Rhod PE LUVs ***36*** were prepared similarly using DSPC **4** (8.0 mg, 9.9 μmol) and 16:0 Liss Rhod PE **34** (20 μL of a 4.4 mM solution in $CHCl_3$, 0.10 μmol).

DOPC LUVs ***38*** were prepared similarly using DOPC **37** (8 mg, 10 μmol). Hydration and extrusion were performed at 40 ºC and rt, respectively.

DSP$_S$C:DSPC (1:2) LUVs ***40*** were prepared similarly using DSP$_S$C **3** (2.0 mg, 2.5 μmol) and DSPC **4** (4.0 mg, 5 μmol). Hydration and extrusion were performed at 65 ºC.

DSP$_S$C:DSPC (2:1) LUVs ***41*** were prepared similarly using DSP$_S$C **3** (4.0 mg, 5 μmol) and DSPC **3** (2.0 mg, 2.5 μmol). Hydration and extrusion were performed at 65 ºC.

4.2. Doxorubicin encapsulated LUVs

DSP$_S$C LUVs ***5*** *⊃ DOX.* As described in reference S12. A thin lipid film was obtained by evaporating a solution of DSP$_S$C **3** (8 mg, 10 μmol) in MeOH/$CHCl_3$ 1:1 (1 mL) on a rotary evaporator and then drying it *in vacuo* overnight. The resulting film was hydrated with a buffer (1.0 mL, 0.3 M citrate buffer, pH 4.0) at 65 °C for 60 min, subjected to freeze (liquid N_2)-thaw (65 °C)

cycles (10x) and extrusions (15x, 65 °C) through a polycarbonate membrane (pore size: 100 nm). The pH of the prepared liposome solution was adjusted to pH 6.5- 7.0 with Na_2CO_3 aqueous solution (0.5 M). A DOX solution (50.0 µL of 1.80 mM in 155 mM NaCl) was preheated at 65 °C and added to the liposome solution over 20 min at 65 °C. Final conditions: ~6 mM lipids; inside: DOX 50 mM, citrate buffer pH 4.0; outside: Na_2CO_3-citrate buffer, pH 6.5- 7.0.

*DSPC LUVs **6** ⊃ DOX* were prepared similarly using DSPC **4** (8 mg, 10 µmol).

*DSP$_S$C LUVs **16a** ⊃ DOX.* Thiosulfonate **13** (8 eq) was added to DSP$_S$C LUVs **5** ⊃ DOX (450 µM, 500 µL) and stirred for 30 min. After, LUVs were purified by size exclusion chromatography using PD MidiTrap G-25 (1 mL buffer). The LUVs concentration was adjusted based on the quantity of encapsulated doxorubicin, following the procedure outlined in section 4.2.2.

*DSP$_S$C LUVs **16b** ⊃ DOX* were prepared similarly using thiosulfonate **14** instead of **13**.

*DSP$_S$C LUVs **16c** ⊃ DOX* were prepared similarly using thiosulfonate **15** instead of **13**.

4.2.1. Detection of encapsulated doxorubicin

General procedure. A stirred dispersion of DSP$_S$C LUVs **5** ⊃ DOX or DSPC LUVs **6** ⊃ DOX (6-7.5 µM lipids) in buffer (2.0 mL, 5 mM HEPES, 172 mM NaCl, pH 7.4) was placed in a disposable PMMA cuvette at 20 °C. Emission spectra were recorded upon excitation at 480 nm with excitation/emission slit widths of 5/5 nm after equilibration for ≈5 min at the same temperature. Emission spectra were recorded after the addition of a solution of Triton X-100 (20 µL of 1.2% in H_2O) and an equilibration of ≈5 min to monitor the recovery of DOX emissions after release from LUVs. As DOX was self-quenched within the LUVs, ~10-fold increase in emission was observed following the destruction of the LUVs by Triton X-100, demonstrating the effective encapsulation of DOX within the LUVs. The emission intensity after disintegration of LUVs was used to correct the concentration of surface-modified LUVs ⊃ DOX **16** after size exclusion chromatography (PD MidiTrap G-25) purification (as described in section 4.2.2.).

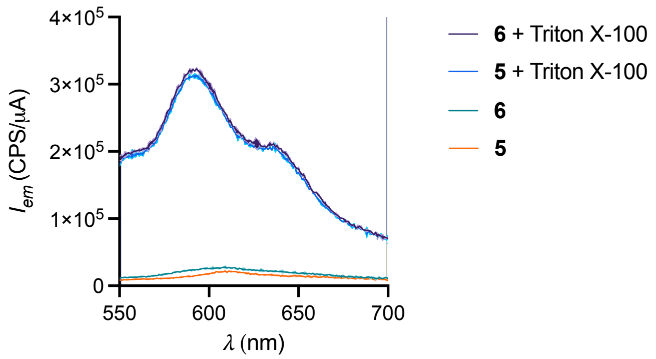

Fig. S7 Emission spectra (λ_{ex} = 480 nm) of DOX in DSPsC LUVs **5** ⊃ DOX or DSPC LUVs **6** ⊃ DOX before (orange for DSPsC LUVs **5** ⊃ DOX, green for DSPC LUVs **6** ⊃ DOX) or after (blue for DSPsC LUVs **5** ⊃ DOX, purple for DSPC LUVs **6** ⊃ DOX) addition of 1.2% Triton X-100 solution.

4.2.2. LUVs ⊃ DOX purification

Efficiency of purification. To evaluate purification, DSPsC LUVs **5** ⊃ DOX (6 µM in HEPES buffer), prepared as described in section 4.2., were analyzed using the same procedure as described in section 4.2.1, with and without size exclusion chromatography. Emission spectra (550–700 nm) were recorded after the addition of Triton X-100 (Figure S8a).

Non-purified, non-modified DSPsC LUVs **5** ⊃ DOX served as the standard. The concentration of purified LUVs ⊃ DOX was determined by normalizing emission intensities to this standard.

DSPsC LUVs **16b** ⊃ DOX, prepared as described in section 4.2, were used as a model to investigate the potential impact of surface modification on purification. The emission intensity of purified DSPsC LUVs **16b** ⊃ DOX was compared to that of non-purified DSPsC LUVs **5** ⊃ DOX.

The recovery after purification was considered quantitative, with less than 5% difference in concentration observed (Figure S8b).

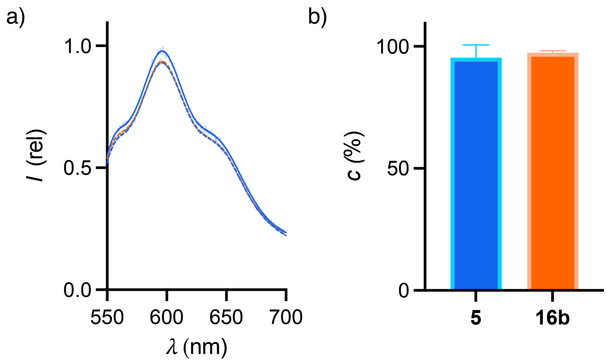

Fig. S8 (a) Normalized emission spectra to DSPsC LUVs **5** ⊃ DOX (blue) of DSPsC LUVs **5** ⊃ DOX after size exclusion chromatography purification (dash blue) and DSPsC LUVs 1**6b** ⊃ DOX after size exclusion chromatography purification (dash orange), after addition of Triton X-100. (b) Amount of DSPsC LUVs **5** ⊃ DOX (blue) or DSPsC LUVs 1**6b** ⊃ DOX (orange) recovered after purification (± SD).

4.3. E4P-Flipper LUVs preparation for cellular uptake

E4P-Flipper **12** (1.70 µM) was added to a preheated solution of empty DSPsC LUVs **5**, or DSPC LUVs **6** (60 °C, 225 µM in HEPES buffer), and the mixture was incubated for 10 min at the same temperature, followed by a cooling to 30 °C over 15 min. The incorporation is presumed to be complete.

5. LUVs characterization

5.1. Dynamic light scattering and zeta potential

For dynamic light scattering (DLS) and zeta potential,[S13] Malvern Zetasizer Nano ZS instrument was used to measure the LUVs' size and electrophoretic mobility. DSPsC LUVs **5** or DSPC LUVs **6** (100 µM in 0.01 M NaCl) were analyzed in batch mode at 25 °C using a folded capillary zeta cell. DLS measurements (size, polydispersity index, and zeta potential) were done in triplicate.

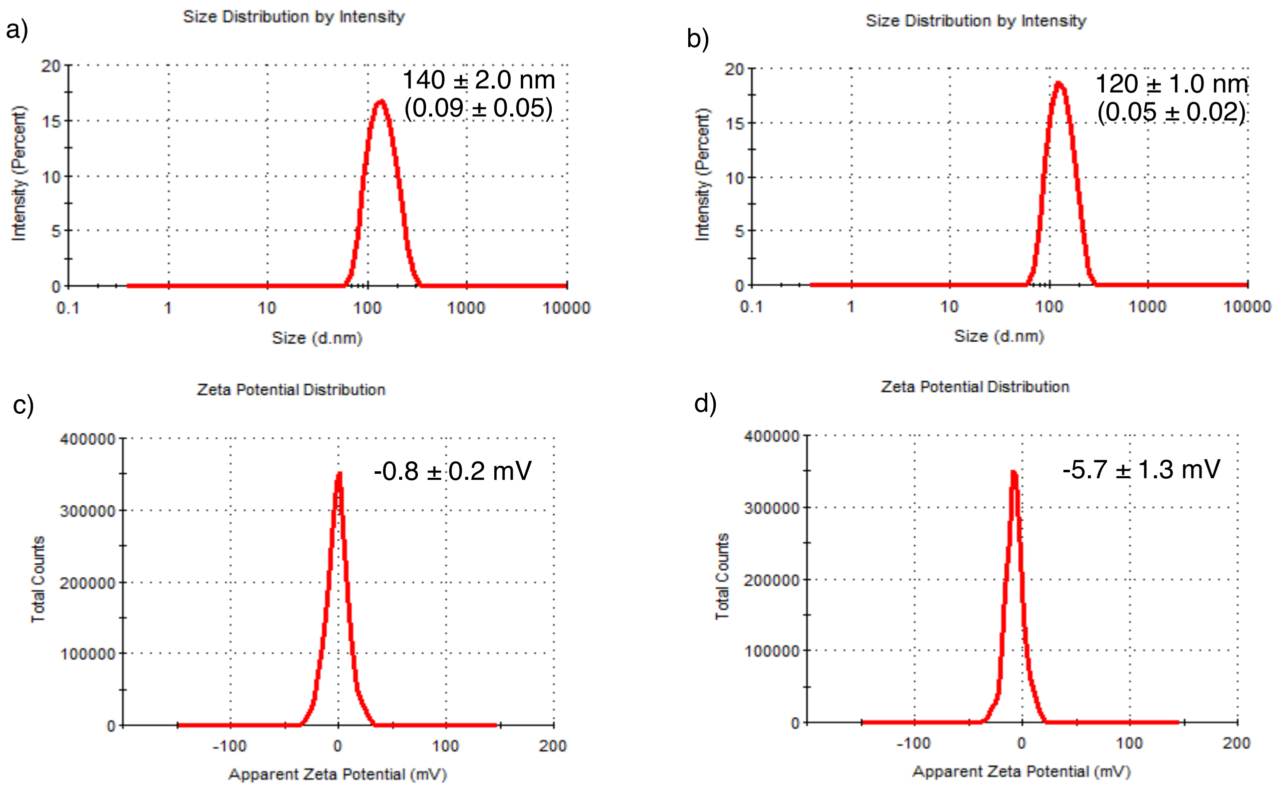

Fig. S9 Results of the dynamic light scattering measurement of the size distribution by intensity (average size reported with SD and polydispersity index reported in parentheses with SD) of DSPC LUVs **6** (a), or DSP_SC LUVs **5** (b). Zeta potential distribution (average zeta potential reported with SD) of DSPC LUVs **6** (c), or DSP_SC LUVs **5** (d).

5.2. Surface modification quantification by DTNB assay

Calibration procedure. A solution of Ac-Cys-OMe **30** in HEPES buffer (0.1-1 eq., 7 µL, pH 7.4) was added to a solution of DTNB **39** in HEPES buffer (7 µL of 10 mM stock solution in 993 µL HEPES buffer, pH 7.4). The absorption spectra were collected between 300 and 540 nm after stirring for 10 min at rt. From the collected spectra, the product of the division of absorption at 432 nm (Abs_{432}) and 324 nm (Abs_{324}) was plotted against the concentration of Ac-Cys-OMe **30** added.

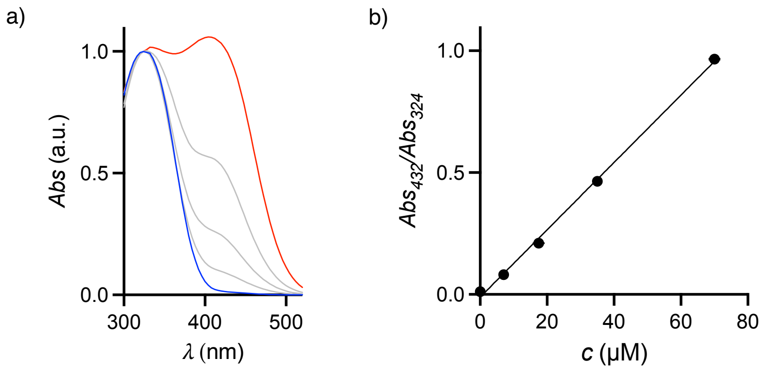

Fig. S10 (a) Normalized absorption spectra of DTNB (70 μM) mixed with Ac-Cys-OMe (0, 0.1, 0.2 0.5, 1 eq., blue to red). (b) The calibration curve ($R^2 > 0.99$) correlating the ratio Abs_{432}/Abs_{324} to the concentration of Ac-Cys-OMe (black filled circles, fit to the linear function, $R^2 > 0.99$).

The concentration of thiol was determined using equation S1.

$$x = \frac{\frac{Abs_{432}}{Abs_{324}} - 0.011}{0.014} \qquad \text{S1}$$

General Procedure. To a solution of empty LUVs (DSP_SC **5** or DSPC **6**, 70 μM), prepared as described in section 4.1., in buffer (1.0 mL, 5 mM HEPES, 172 mM NaCl, pH 7.4), was added a solution of DTNB **39** (1 eq., 7 μL of 10 mM in HEPES buffer pH 7.4). The absorption spectra were collected between 300 and 540 nm after stirring for 10 min at rt.

Surface-modified LUVs. The surface-modified $DSP_{S}C$ LUVs **16** were prepared following the procedure described in section 4.2. using empty $DSP_{S}C$ LUVs **5**. Resulting LUVs were treated with DTNB **39** following the general procedure. It was presumed that the recovery of LUVs would be complete based on the efficiency of purification described in section 4.2.2.

Table S1 Concentration of phosphorothioate of empty LUVs with or without surface modification.

Entry	LUVs	Conc. (µM)[a]
1	**5**	37 ± 1
2	**6**	-
3	**16a**	< 1
4	**16b**	4 ± 1
5[b]	**16b**	5 ± 1
6	**16c**	3 ± 1

[a]Results from Figure S11a-b using equation S1, concentration of free phosphonothioate reacting with DTNB. Duplicate measurements were conducted and presented with standard deviation (± SD). [b]Results from Figure S11c using DSP$_S$C LUVs **16b** ⊃ DOX, recovered concentration after purification (97 ± 1%) was measured using the procedure described in section 4.2.2.

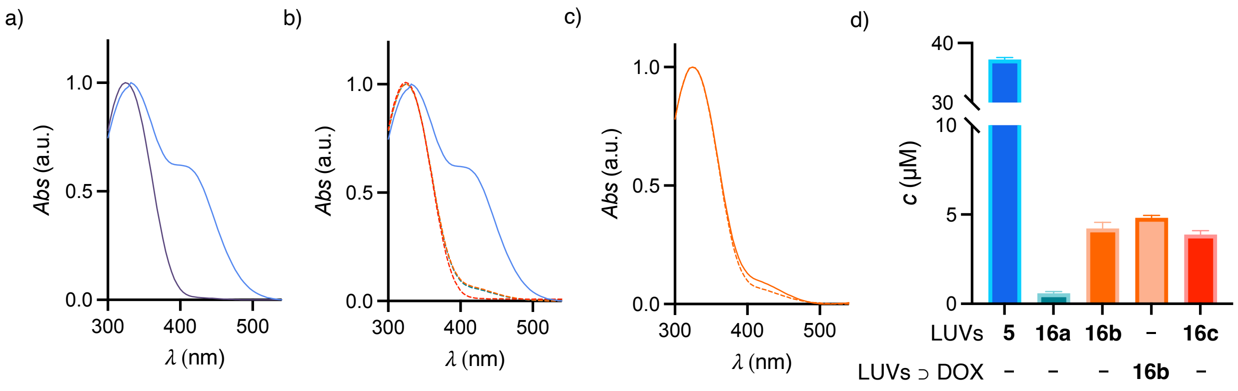

Fig. S11 Normalized absorption spectra of DTNB after the addition of (a) DSP$_S$C LUVs **5** (blue), DSPC LUVs **6** (purple), or (b) DSP$_S$C LUVs **5** (blue), DSP$_S$C LUVs **16a** (dash green), DSP$_S$C LUVs **16b** (dash orange), and DSP$_S$C LUVs **16c** (dash red). (c) Normalized absorption spectra of DTNB after the addition of DSP$_S$C LUVs **16b** (dash orange) or DSP$_S$C LUVs **16b** ⊃ DOX (orange). (d) Calculated concentration of thiol (± SD) using equation S1 of DSP$_S$C LUVs **5** (blue), DSP$_S$C LUVs

16a (green), DSP$_S$C LUVs **16b** (orange), DSP$_S$C LUVs **16b** ⊃ DOX (light orange), and DSP$_S$C LUVs **16c** (red).

5.3. Flipper properties in LUVs

General procedure. As described in reference S11. A solution of the probe **11** or **12** (2.0 µL of a stock solution in DMSO) was added to a stirred dispersion of DSP$_S$C LUVs **5**, or DSPC LUVs **6** (75 µM) in buffer (2.0 mL, 5 mM HEPES, 172 mM NaCl, pH 7.4) at 65 °C in a disposable PMMA cuvette. After equilibration for ≈20 min at the same temperature, the sample was cooled to the desired temperature, and the excitation spectra were recorded after equilibration for ≈10 min. Excitation spectra were recorded for emission at 650 nm with excitation/emission slit widths of 5/20 nm, and a long-pass filter (cut on wavelength 540 nm) in the emission path. Spectra obtained under the same conditions without probes but with DMSO (2.0 µL) were used as backgrounds and subtracted from the spectra of probes. Average spectra of four consecutive scans are plotted with Lowess smoothing for noisy spectra.

5.3.1. Concentration dependence

Following the general procedure, excitation spectra were recorded at 30 °C after the addition of various concentrations of probes **11** or **12** (in DMSO, 2.0 µL of 0–2000 µM) to DSP$_S$C LUVs **5**, or DSPC LUVs **6** (75 µM) in buffer (2.0 mL, 5 mM HEPES, 172 mM NaCl, pH 7.4) at 60 °C. After equilibration for ≈20 min at the same temperature, the sample was cooled to the desired temperature, and the excitation spectra were recorded after equilibration for ≈10 min

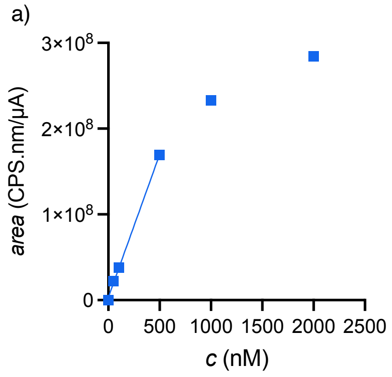

Fig. S12 Dependence of excitation intensity (integrated area of excitation spectra λ_{ex} 350-580 nm) on the concentration of flipper **11** or **12** (0-2000 µM) on LUVs **5** or **6** (75 µM) at 30 °C. (a, b) Flipper probes **12** in DSPsC LUVs **5** (a, 0-2000 nM) or DSPC LUVs **6** (b, 0-500 nM), and (c) flipper probes **11** in DSPsC LUVs **5** (0-100 nM) (blue slope, R^2 > 0.99). Linear concentration dependence of the excitation intensity was found with flipper probes **12** in both DSPsC LUVs **5** and DSPC LUVs **6** up to ~500 nM, while only up to ~10 nM with **11** in DSPsC LUVs **5**.

5.3.2. Temperature dependence

Following the general procedure, excitation spectra were recorded at lowering temperatures from 70 to 20 °C after adding probe **11** (in DMSO, 2.0 µL, 10 nM) at 70 °C. Three spectra were recorded at each temperature after >5 min of equilibration and averaged. The temperature dependence of spectra was concentration-independent (10-50 nM) at 20-70 °C.

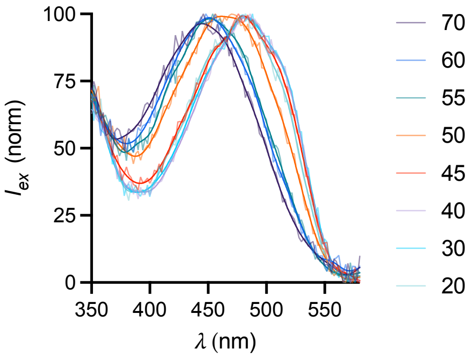

Fig. S13 Representative normalized excitation spectra (λ_{em} = 650 nm) of flipper probes **11** (10 nM) in DSPsC LUVs **5** (phase transition 48 ± 1 °C), at 70–20 °C.

5.3.3. Extraction of 11 or 12 with BSA from LUVs

As described in reference S14. Following the general procedure (section 5.2.), excitation spectrum of an equilibrated solution of **11** or **12** (in DMSO, 2.0 µL of 5 or 500 nM) in buffer (2.0 mL, 5 mM HEPES, 172 mM NaCl, pH 7.4) containing DSP$_S$C LUVs **5** or DSPC LUVs **6** (75 µM) was recorded (λ_{em} = 650 nm, 5 nm slit) at 30 °C. Then, increasing volume of BSA (60 to 120 µM) was added to the solution, and excitation spectra were recorded 2 min after each addition. Excitation spectra (λ_{em} = 650 nm) of LUVs in buffer solution (2.0 mL, 5 mM HEPES, 172 mM NaCl, pH 7.4) and BSA (0 to 120 µM) at 30 °C were recorded to serve as background. The partitioning of probes **11** or **12** between the buffer and the membranes of LUVs was demonstrated by their interaction with external bovine serum albumin (BSA). A poor extraction by BSA indicated a better partitioning of the probe within the bilayer membranes.

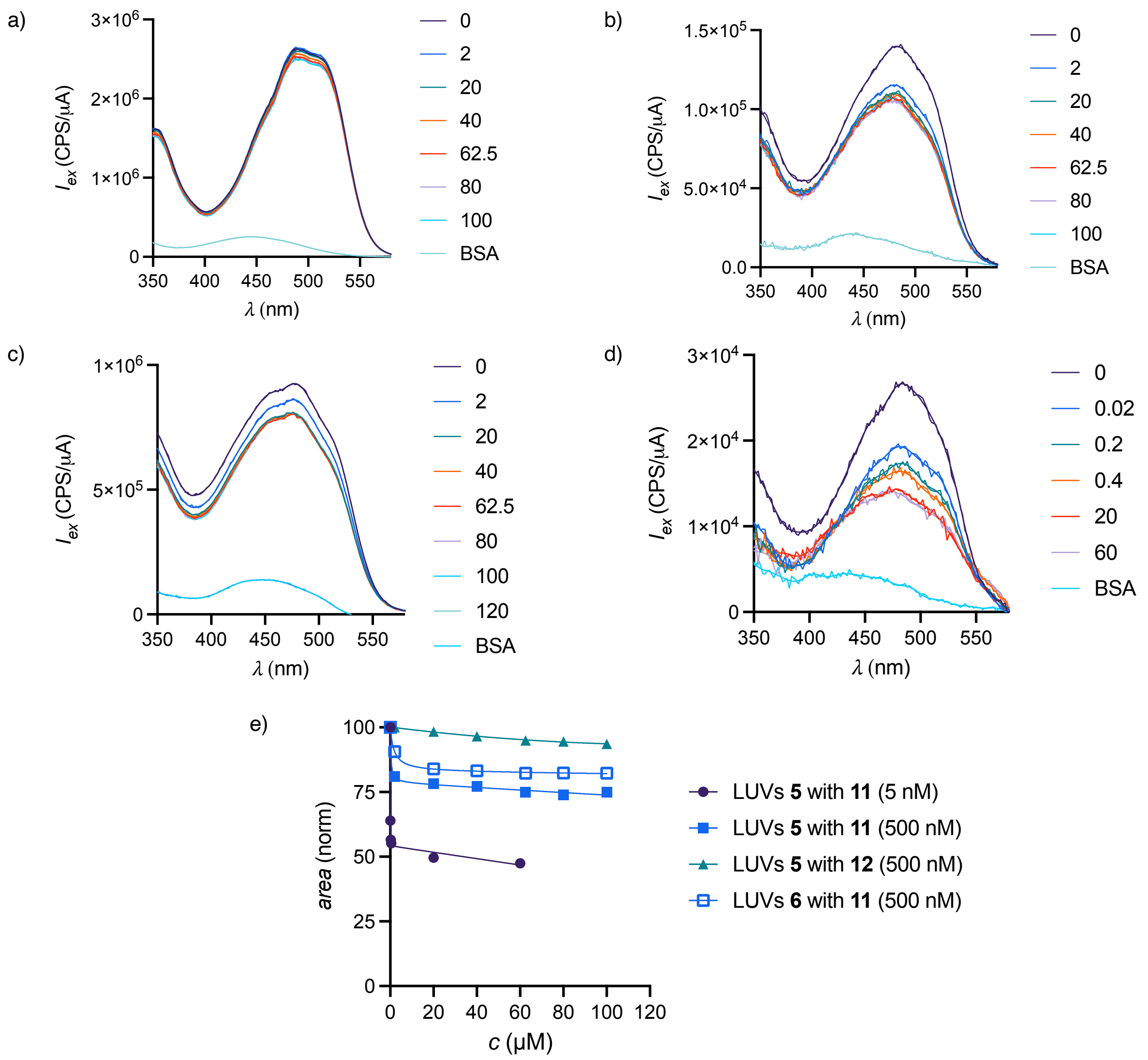

Fig. S14 Representative excitation spectra (λ_{em} = 650 nm) of flipper probes (a) **12** or (b-d) **11** (a-c, 500 nM; d, 5 nM) in (a, b, d) DSPsC LUVs **5** or (c) DSPC LUVs **6** (75 µM) with varying concentrations of BSA (0–120 µM) at 30 °C. (e) Normalized area of integrated excitation spectra (λ_{ex} 350-580 nm). *Area = 0*: the flipper probe in BSA without LUVs (cyan in a-d), *area = 100*: the flipper probe in LUVs without BSA (purple in a-d).

Table S2 Properties of flipper **11** or **12** in LUVs.

Entry	LUVs	Flipper	Max. Conc. (nM)[a]	Flipper ⊂ LUVs (%)[b]
1	**5**	**11**	10 ± 5	75 ± 2<br>50 ± 5[c]
2	**6**	**11**	-	80 ± 2
3	**5**	**12**	500 ± 50	95 ± 2
4	**6**	**12**	500 ± 50	-

[a]Results from Figure S12, the maximum concentration of flipper probes incorporated into the LUVs' membrane, exhibiting a linear concentration dependence. [b]Results from Figure S14 at 500 nM of flipper probes **11** or **12** showing the remaining amount of flipper in the LUVs membrane at the maximum BSA concentration. [c]Results from Figure S14d of LUVs **5** with 5 nM of flipper **11**.

6. Cell culture

As described in reference S15. Human cervical cancer-derived HeLa Kyoto (HK) cells were cultured in complete DMEM (GlutaMAX, 4.5 g/L D-glucose, phenol red) medium, which contains 10% fetal bovine serum (FBS) and 1% Penicillin/Streptomycin (PS). The cells were grown under 5% CO_2 humidified atmosphere at 37 °C on a 25 cm^3 tissue culture flask (TPD Corporation). Cells were harvested by treatment with 1 mL of phenol-red free TrypLE Express, followed by the addition of 2 mL of complete phenol red free FDMEM (GlutaMAX, 4.5 g/L D-glucose) medium at 37 °C. The cells were plated at 8×10^4 cells/mL (150 μL/well for 96 well plate or 1.0 mL/well for 35 mm glass-bottomed dishes).

7. Evaluation of LUVs uptake by CLSM

7.1. General experimental procedure

As described in reference S15. HK cells were plated and grown on 35 mm glass-bottomed dishes (MatTek Corporation), as described in section 6, and cultured overnight. After removing the medium, the cells were washed with DPBS (3 x 1 mL) and with Leibovitz's L-15 medium (3 x 1 mL)

before being treated with DSPsC LUVs **5** ⊃ DOX, or DSPC LUVs **6** ⊃ DOX solution (75 μM, in 1.0 mL L-15 medium). The cells were incubated for 0.5 to 6 h at 37 °C, then the media was removed by aspiration. Cells were washed with PBS (3 x 1 mL) and with L-15 medium (3 x 1 mL) before being treated with plasma membrane (PM) stain, BioTracker 655 Red Cytoplasmic Membrane (5.0 μL in 1.0 mL L-15 medium). The cells were kept in L-15 medium at room temperature during the microscope experiment. Distribution of fluorescence was analyzed without fixing using a confocal laser scanning microscope (Leica SP8) equipped with 63X oil immersion objective lens.

Images were sequentially recorded at 525 – 620 nm (Leica HyD™ detector) upon excitation at 488 nm (30% laser power) for DOX and at 648 – 714 nm (Leica HyD™ detector) upon excitation at 638 nm (0.7% laser power) for PM stain.

7.2. Data analysis

To analyze and quantify the LUVs ⊃ DOX uptake in cells, Fiji software was used first to segment the image and generate a relevant mask, which was then applied to the fluorescent image to finally extract relevant measurements.

The initial step involves generating a cell mask using the PM staining channel (Figure S6b) to define the plasma membrane region of interest ($ROI_{membrane}$, Figure S6d). Subsequently, the cytoplasm is defined as the region of interest (ROI_{cyto}) using the inner leaflet membrane delimitation (Figure S6e). Intensity per pixel measurements were conducted for DOX channel images within the ROI_{cyto} (Figure S6f). The results obtained for the DSPsC LUVs **5** were compared to those of DSPC LUVs **6**. For each condition, intensity measurements were conducted on a minimum of four cells per image (excluding cells at the edge) and at least three distinct images. The results were reported as the average per image, from technical duplicates, of this measurement with the standard error of the mean (SEM).

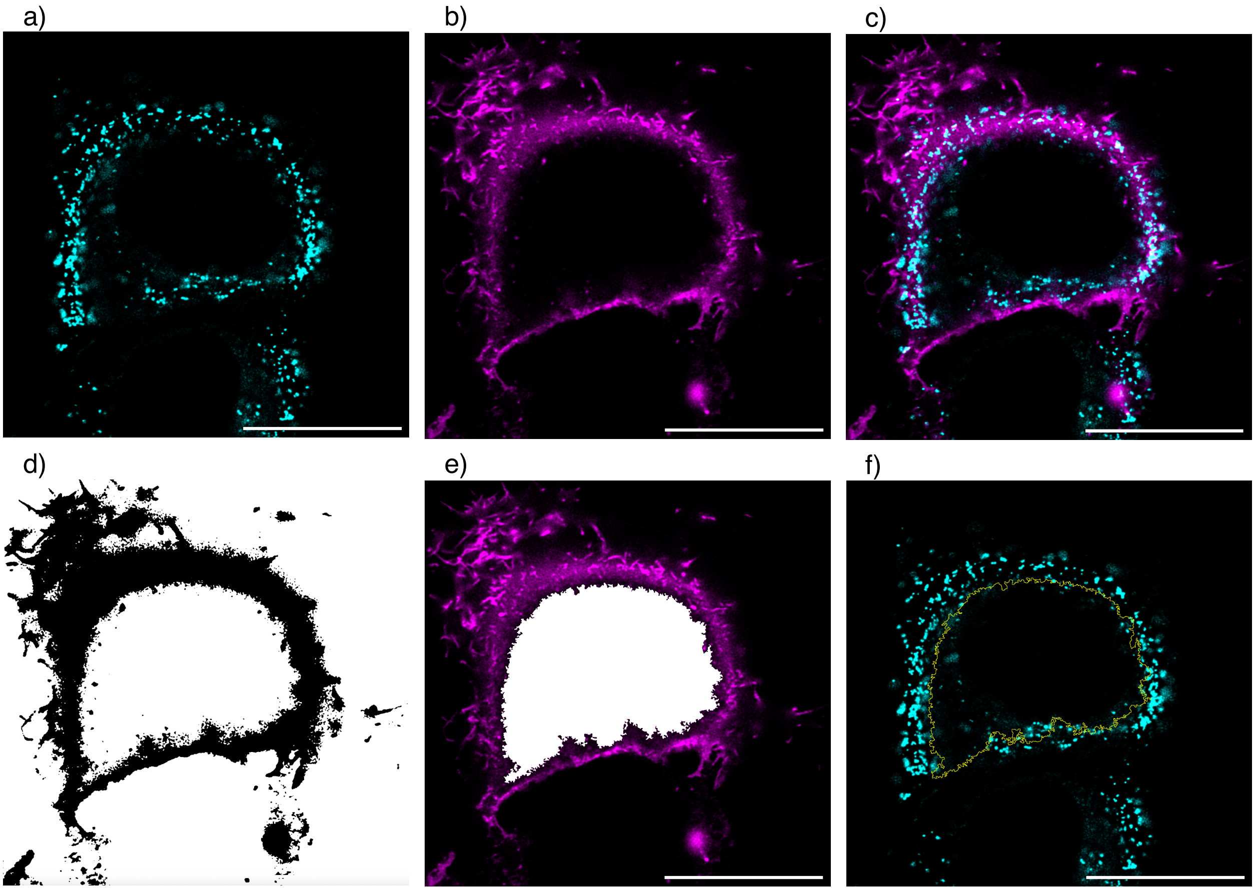

Fig. S15 (a) CLSM image of HK cells treated with DSPC-LUVs **6** ⊃ DOX in DOX channel (cyan). (b) Corresponding image in PM stain channel (magenta). (c) Overlay of CLSM images (a) and (b). (d) Creation of the plasma membrane mask ($ROI_{membrane}$) based on image (b). (e) Application of the mask to select the cytoplasm (ROI_{cyto} in white) on image (b) or (f, ROI_{cyto} bordered in yellow) on image (a). Scale bar 20 µm.

7.3. Results for uptake into HK cells

7.3.1. LUVs ⊃ DOX

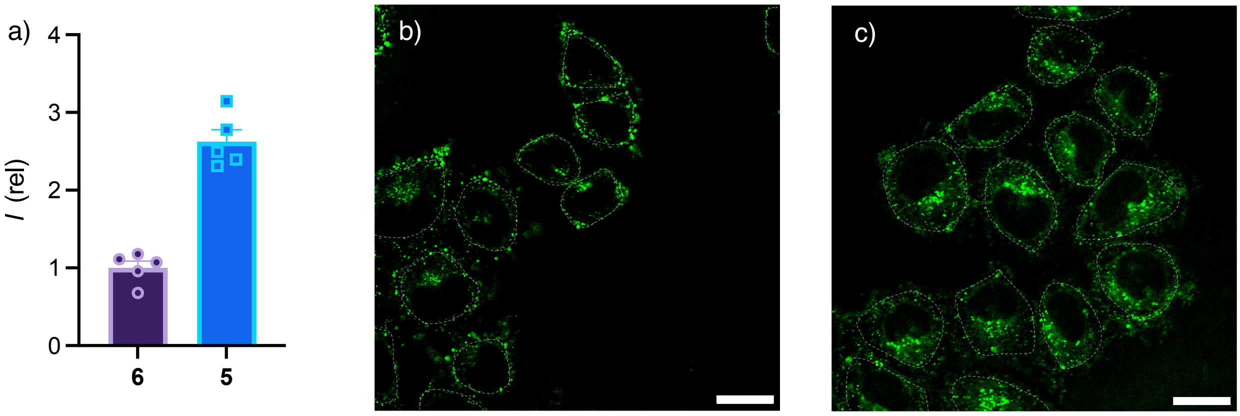

Fig. S16 Uptake of LUVs ⊃ DOX in HK cells after 0.5 h incubation. (a) Relative fluorescence intensity *I* (rel) ± SEM (one point per image) of DOX in ROI_{cyto} of HK cells treated with LUVs ⊃ DOX. CLSM image, same brightness, of HK cells incubated for 30 min with DSPC LUVs **6** ⊃ DOX (b), DSP_SC LUVs **5** ⊃ DOX (c), cell membrane represented in dotted lines. Scale bar 20 µm.

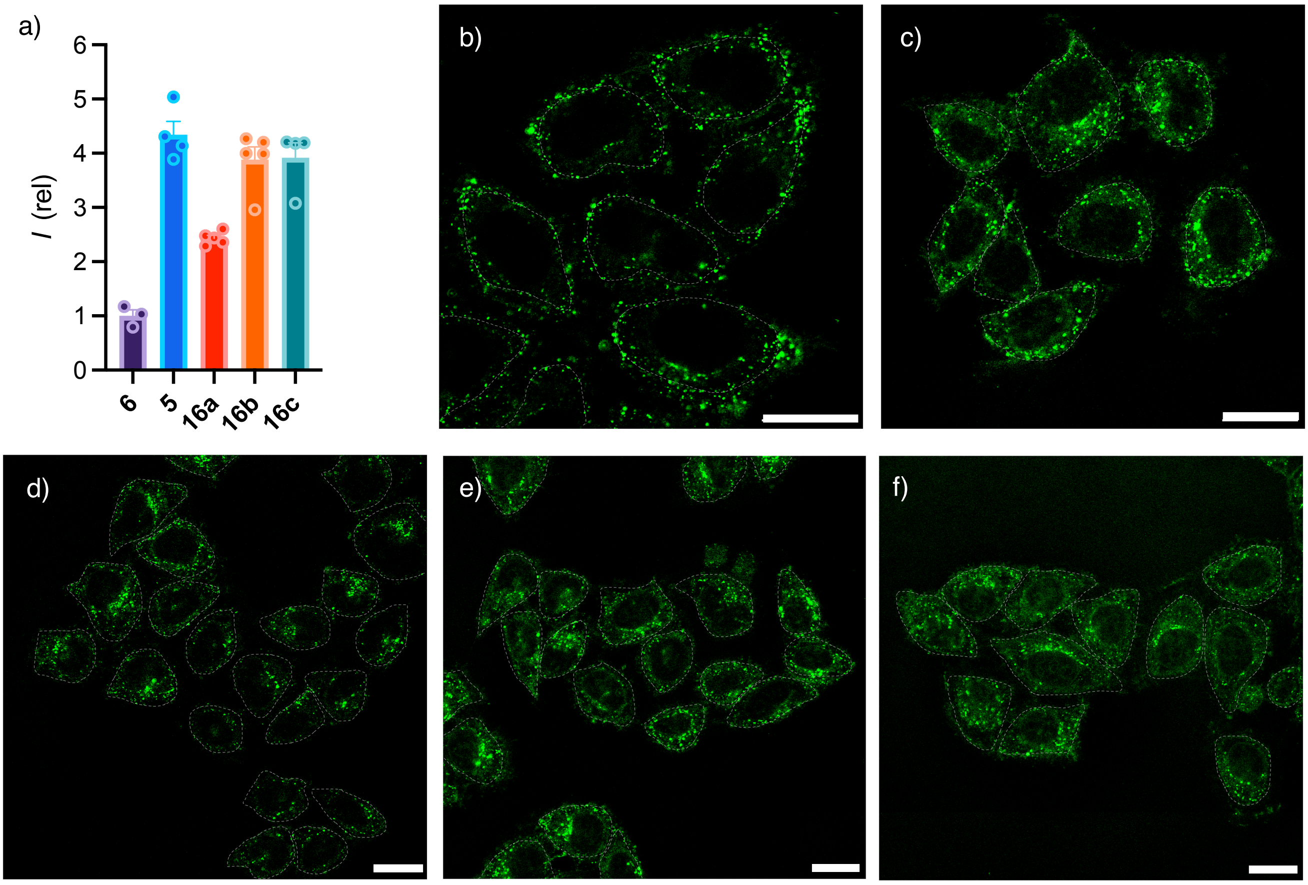

Fig. S17 Uptake of LUVs ⊃ DOX in HK cells after 1 h incubation. (a) Relative fluorescence intensity *I* (rel) ± SEM (one image per point) of DOX in ROI_{cyto} of HK cells treated with LUVs ⊃ DOX. CLSM image, same brightness, of HK cells incubated for 1 h with DSPC LUVs **6** ⊃ DOX (b), DSP$_S$C LUVs **5** ⊃ DOX (c), DSP$_S$C LUVs **16a** ⊃ DOX (d), DSP$_S$C LUVs **16b** ⊃ DOX (e), DSP$_S$C LUVs **16c** ⊃ DOX (f), cell membrane represented in dotted lines. Scale bar 20 µm.

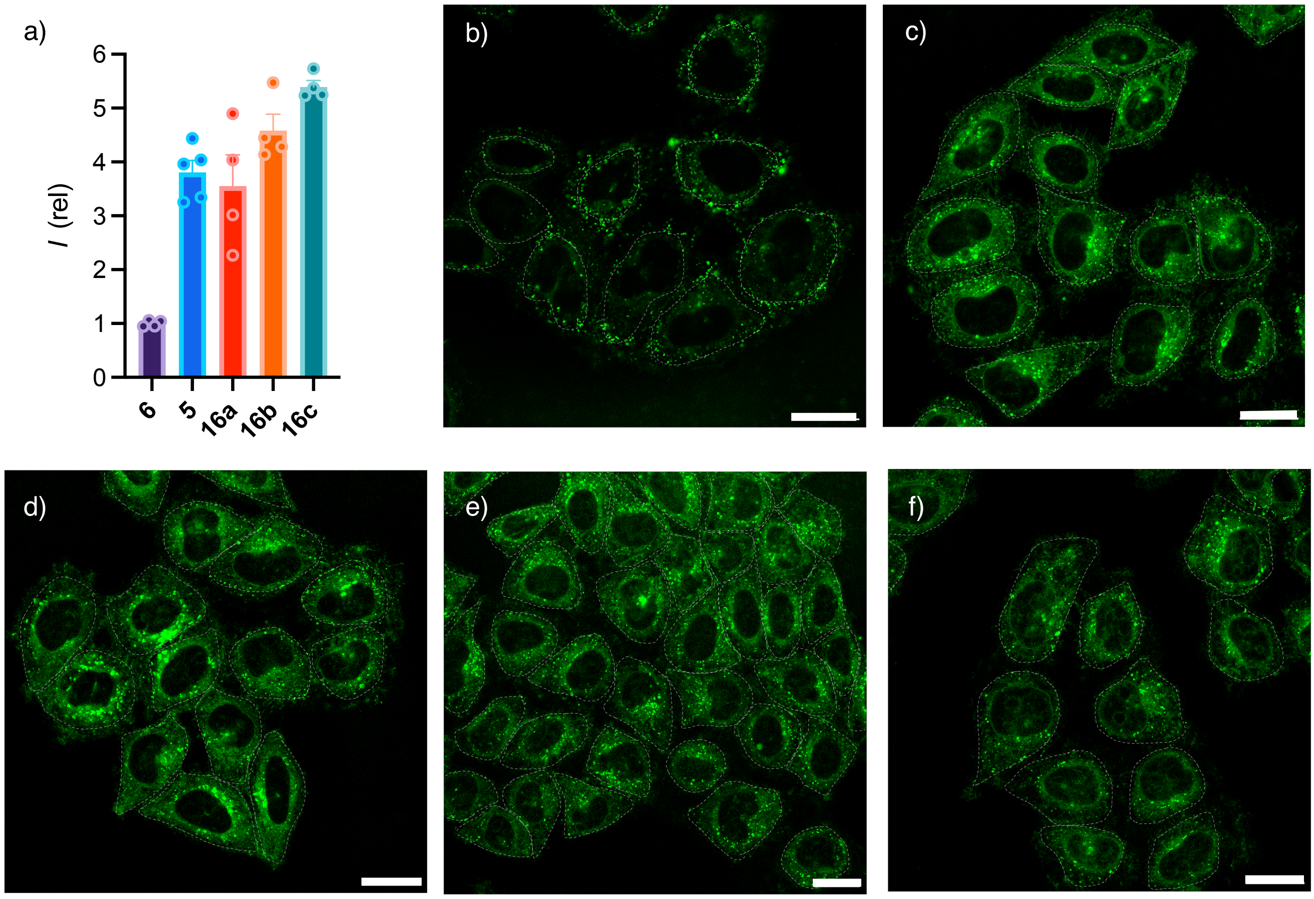

Fig. S18 Uptake of LUVs ⊃ DOX in HK cells after 2 h incubation. (a) Relative fluorescence intensity *I* (rel) ± SEM (one image per point) of DOX in ROI_{cyto} of HK cells treated with LUVs ⊃ DOX. CLSM image, same brightness, of HK cells incubated for 2 h with DSPC LUVs **6** ⊃ DOX (b), DSP_SC LUVs **5** ⊃ DOX (c), DSP_SC LUVs **16a** ⊃ DOX (d), DSP_SC LUVs **16b** ⊃ DOX (e), DSP_SC LUVs **16c** ⊃ DOX (f), cell membrane represented in dotted lines. Scale bar 20 μm.

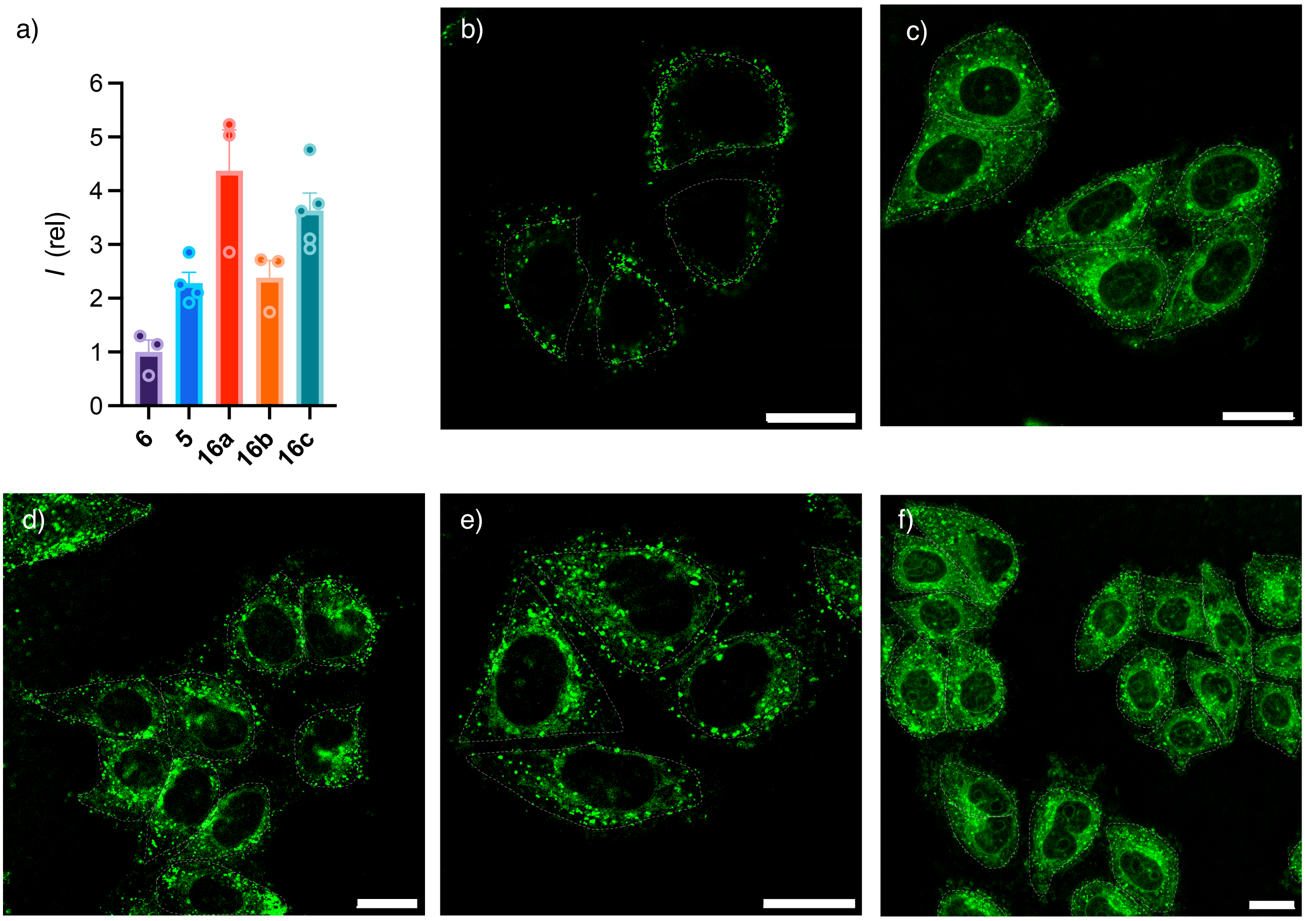

Fig. S19 Uptake of LUVs ⊃ DOX in HK cells after 4 h incubation. (a) Relative fluorescence intensity *I* (rel) ± SEM (one image per point) of DOX in ROI_{cyto} of HK cells treated with LUVs ⊃ DOX. CLSM image, same brightness, of HK cells incubated for 4 h with DSPC LUVs **6** ⊃ DOX (b), DSP_SC LUVs **5** ⊃ DOX (c), DSP_SC LUVs **16a** ⊃ DOX (d), DSP_SC LUVs **16b** ⊃ DOX (e), DSP_SC LUVs **16c** ⊃ DOX (f), cell membrane represented in dotted lines. Scale bar 20 µm.

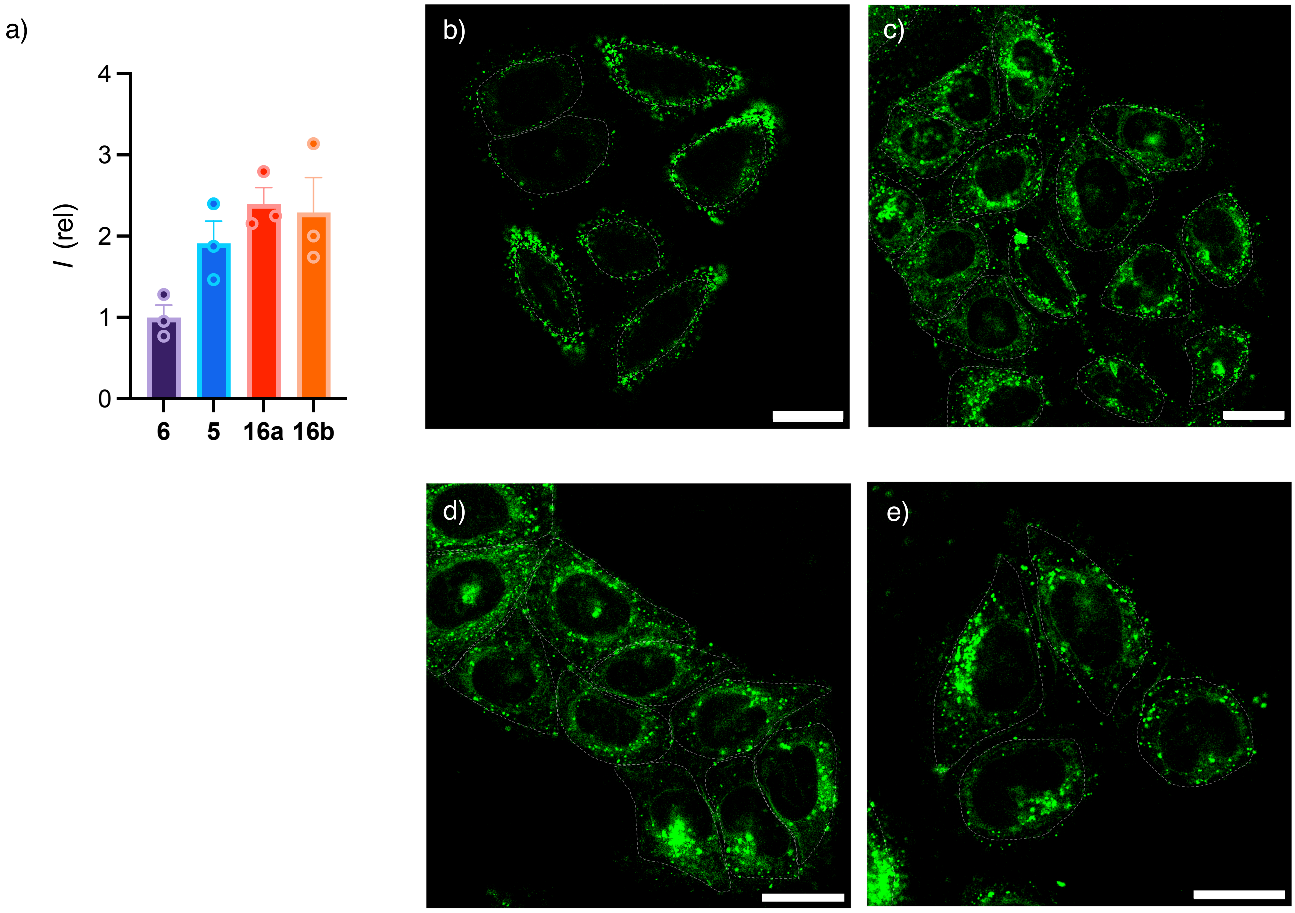

Fig. S20 Uptake of LUVs ⊃ DOX in HK cells after 6 h incubation. (a) Relative fluorescence intensity *I* (rel) ± SEM (one image per point) of DOX in ROI_{cyto} of HK cells treated with LUVs ⊃ DOX. CLSM image, same brightness, of HK cells incubated for 6 h with DSPC LUVs **6** ⊃ DOX (b), DSP_SC LUVs **5** ⊃ DOX (c), DSP_SC LUVs **16a** ⊃ DOX (d), DSP_SC LUVs **16b** ⊃ DOX (e), cell membrane represented in dotted lines. Scale bar 20 μm.

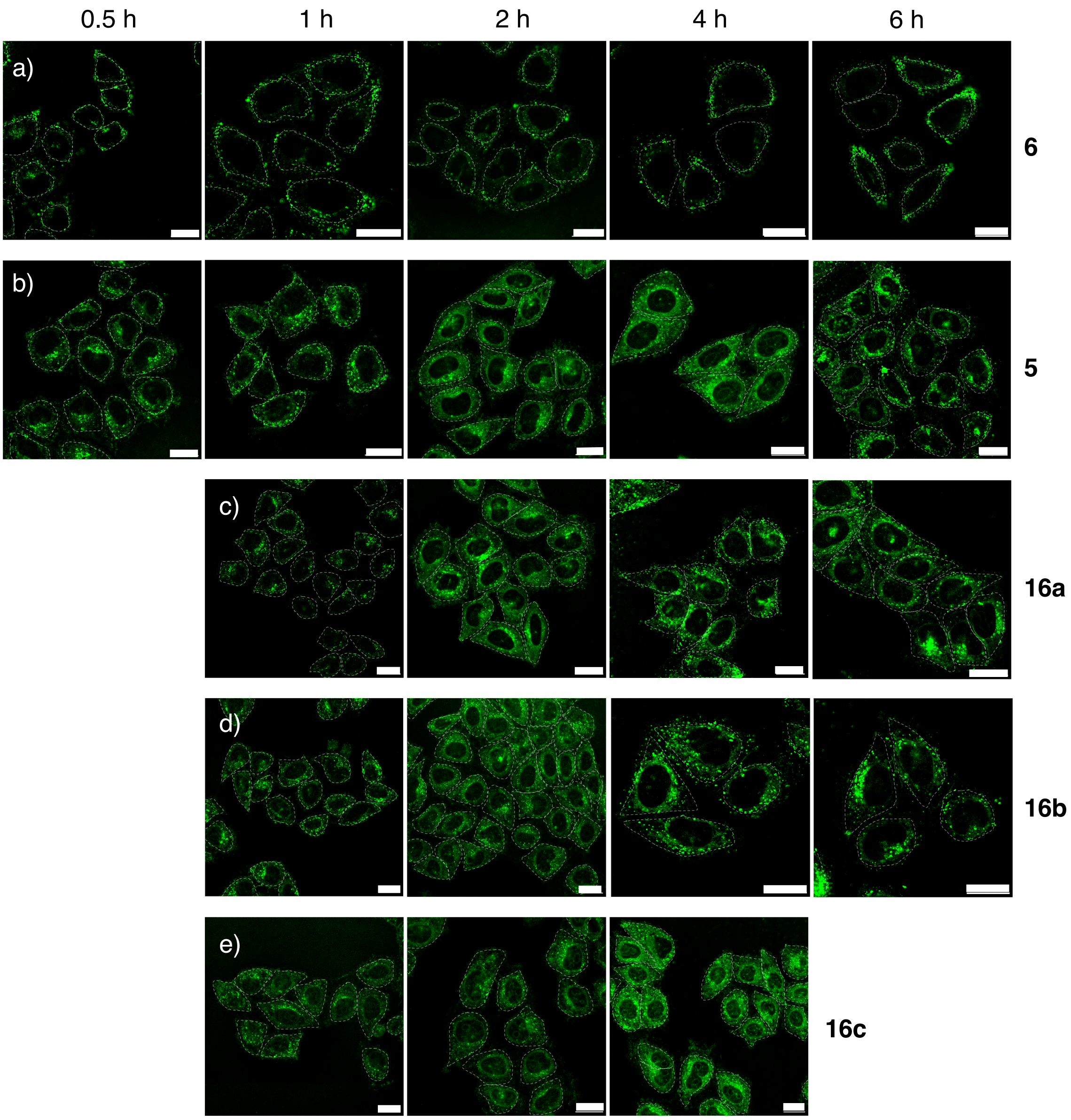

Fig. S21 CLSM image (63x) of uptake time course (0.5, 1, 2, 4, or 6 h incubation) of DSPC LUVs **6** ⊃ DOX (a), DSP$_S$C LUVs **5** ⊃ DOX (b), DSP$_S$C LUVs **16a** ⊃ DOX (c), DSP$_S$C LUVs **16b** ⊃ DOX (d), DSP$_S$C LUVs **16c** ⊃ DOX (e) in HK cells, cell membranes represented in dotted lines. Images are from Figures S16-S20. Scale bar 20 μm.

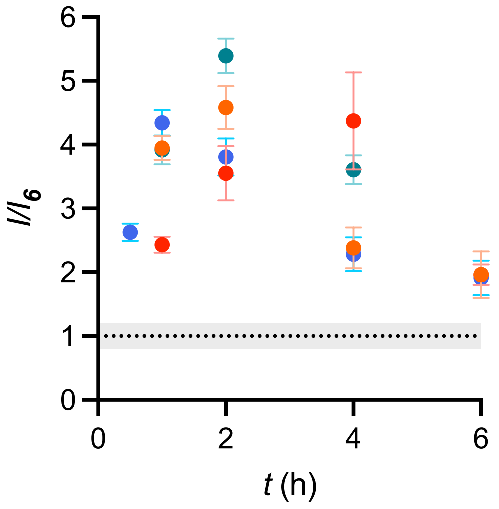

Fig. S22 Resulting fluorescence intensity I (rel) ± SEM normalized to DSPC LUVs **6** ⊃ DOX from 0.5 to 6 h incubation of (●) DSPsC LUVs **5** ⊃ DOX, (●) DSPsC LUVs **16a** ⊃ DOX, (●) DSPsC LUVs **16b** ⊃ DOX, (●) DSPsC LUVs **16c** ⊃ DOX (SEM of DSPC LUVs **6** ⊃ DOX represented as a grey band).

7.3.2. LUVs ⊃ E4P-Flipper

HK cells were plated and grown on 35 mm glass-bottomed dishes (MatTek Corporation), as described in section 5, and cultured overnight. After removing the medium, the cells were washed with DPBS (3 x 1 mL) and with Leibovitz's L-15 medium (3 x 1 mL) before being treated with DSPsC LUVs **5** ⊃ E4P-Flipper **12**, or DSPC LUVs **6** ⊃ E4P-Flipper **12**, or DSPsC:DSPC (1:2) LUVs **40** ⊃ E4P-Flipper, or DSPsC:DSPC (2:1) LUVs **41** ⊃ E4P-Flipper (lipid: 75 µM, **12**: 0.5 µM, in 1.0 mL L-15 medium), or E4P-Flipper solution (0.5 µM, in 1.0 mL L-15 medium). The cells were incubated for 0.5 to 6 h at 37 °C, then the media was removed by aspiration. Cells were washed with PBS (3 x 1 mL) and with L-15 medium (3 x 1 mL). The cells were kept in L-15 medium at room temperature during the microscope experiment. Distribution of fluorescence was analyzed without fixing using a confocal laser scanning microscope (Leica SP8) equipped with 63X oil immersion objective lens.

Images were recorded at 550 – 650 nm (Leica HyD™ detector) upon excitation at 488 nm (10% laser power).

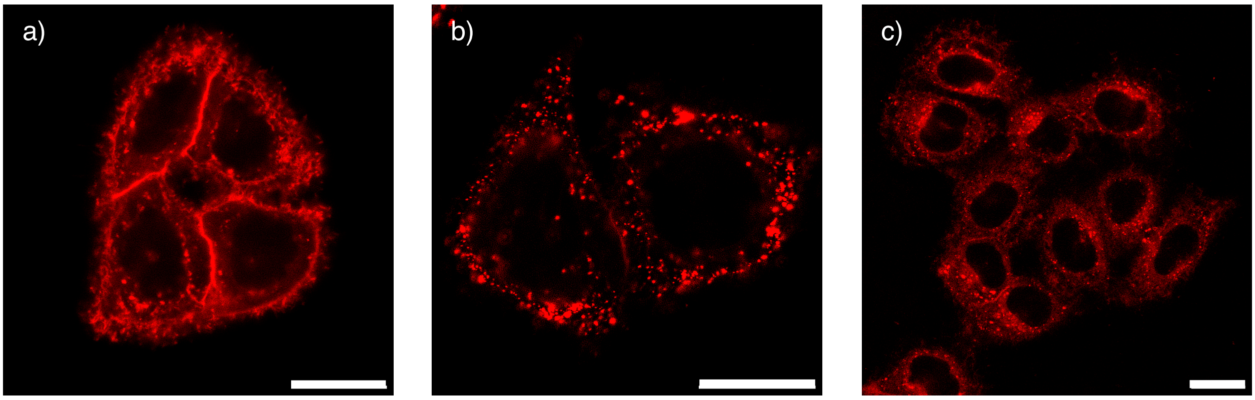

Fig. S23 CLSM image of HK cells incubated for 2 h with E4P-Flipper **12** alone (a), or embedded in DSPC LUVs **6** ⊃ E4P-Flipper (b), or DSPsC LUVs **5** ⊃ E4P-Flipper (c). Scale bar 20 µm.

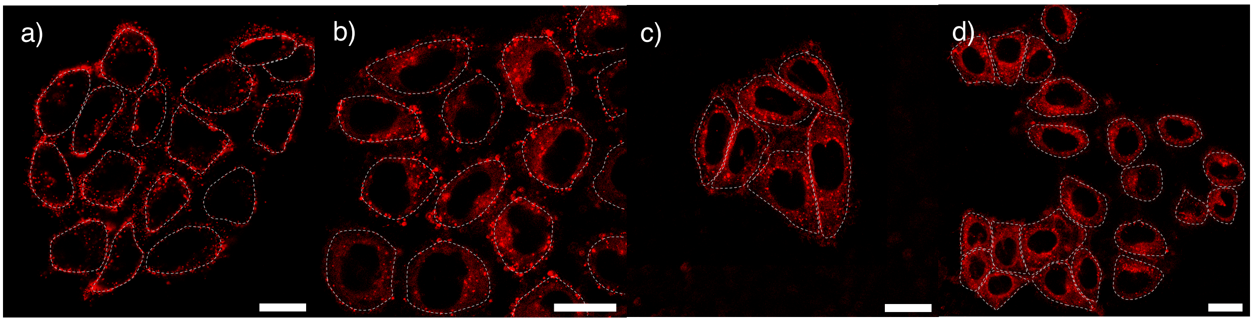

Fig. S24 CLSM image of HK cells incubated for 2 h with DSPC LUVs **6** ⊃ E4P-Flipper (a), or DSPsC:DSPC (1:2) LUVs **40** ⊃ E4P-Flipper (b), or DSPsC:DSPC (2:1) LUVs **41** ⊃ E4P-Flipper (c), or DSPsC LUVs **5** ⊃ E4P-Flipper (d), cell membranes represented in dotted lines. Scale bar 20 µm.

8. AHCHT uptake inhibition assays

8.1. General procedure for AHCHT imaging LUVs ⊃ DOX uptake in HK cells

As described in reference S15. HK cells were plated and grown on a 96 well plate, as described in section 5, and cultured overnight. Then medium was removed, and cells were washed with PBS (3

× 3 mL/well) followed by fresh L-15 serum-free medium (4 × 100 µL/well) using a plate washer (Biotek EL406®), and kept in a 100 µL of the latter medium. The solution of inhibitor (1-10 mM, DMSO) was diluted in L-15 medium to give a solution at 3x final concentration, of which 50.0 µL was added to each well, resulting in a final volume of 150 µL per well. The cells were incubated under a 5% CO_2 humidified atmosphere at 37 °C for 60 min.

With CAX inhibitors. Afterward, to remove the excess of inhibitor, the cells were washed with PBS (2 x 3 mL), and the medium was exchanged with L-15 medium (4 x 200 µL), keeping a final volume of 100 µL/well. A solution of LUVs ⊃ DOX **5** or **6** (6 mM in buffer, Na_2CO_3-citrate buffer, pH 6.5- 7.0) was diluted in L-15 medium to give a solution at 3x final concentration (225 µM), of which 50.0 µL was added to the well resulting in a final volume of 150 µL per well (75 µM). The cells were incubated under 5% CO_2 humidified atmosphere at 37 °C for 120 min.

With endocytosis inhibitors. After the incubation with inhibitors, the cells were washed with PBS (2 x 3 mL), and the medium was exchanged with L-15 medium (4 x 200 µL), keeping a final volume of 100 µL/well. A solution of LUVs ⊃ DOX **5** or **6** (6 mM in buffer, Na_2CO_3-citrate buffer, pH 6.5- 7.0), and a solution of inhibitor (1-10 mM, DMSO) were diluted in L-15 medium to give solutions at 4x final concentration, of which 50.0 µL of each were added to the well resulting in a final volume of 200 µL per well. The cells were incubated under 5% CO_2 humidified atmosphere at 37 °C for 120 min.

Afterward, the cells were washed with PBS and the medium was exchanged with L-15 medium keeping a final volume of 100 µL/well, and a solution of Hoechst 33342 (100 µg/mL) in L-15 medium (50.0 µL/well) was added. After 15 min of incubation under 5% CO_2 humidified atmosphere at 37 °C, cells were washed with PBS (3 × 3 mL/well) and kept in L-15 medium (100 µL/well) for live cell imaging. The distribution of fluorescent signals was captured on a IXM-C automated microscope in widefield mode (10X) with two channels (blue/yellow): blue channel (377/50 nm excitation filter; 477/60 nm emission filter, for Hoechst 33342), and yellow channel

(531/40 nm excitation filter; 593/40 nm emission filter, for LUVs ⊃ DOX). The rest of the parameters were adjusted according to the nature of the experiment. Duplicates were performed for each condition.

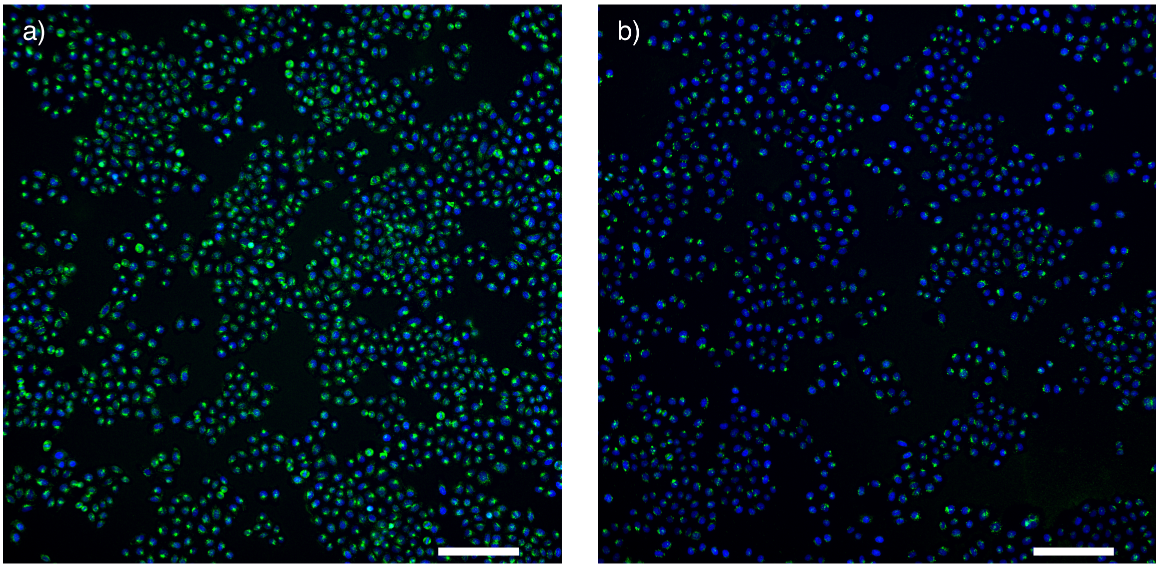

Fig. S25 Representative microscopic images (10X) with the same brightness of DSP$_S$C LUVs **5** ⊃ DOX (a) without and (b) with inhibitor **7** at 500 μM (DSP$_S$C LUVs ⊃ DOX **5** green; blue: Hoechst 33342; scale bar 200 μm).

Control experiments were performed to confirm endocytosis inhibition by replacing LUVs ⊃ DOX with EGF A-647 (200 ng/mL), or Dextran A-647 (2.0 mg/mL). For Dextran A-647, the incubation time of 30 min was used instead of 120 min. The distribution of fluorescent signals was captured on a IXM-C automated microscope widefield (10X) with two channels (blue/red), blue channel (377/50 nm excitation filter; 477/60 nm emission filter, for Hoechst 33342), and red channel (620/50 nm; emission filter: 690/50 nm, for EGF A-647/Dextran A-647).

8.2. Data analysis

Resulting images were automatically analyzed and quantified using a protocol similar to that in reference S15. Briefly, the nuclei and cell bodies were segmented using the blue channel image

(Hoechst 33342). Dividing and dying cells were detected based on their shape factor (round shape, > 0.8) and filtered out. The resulting objects were then grown and all cells in the vicinity were removed from the analysis. All the cells touching the border of the image were removed to prevent inaccurate quantification in the final mask. Finally, fluorescence of the yellow (for LUVs ⊃ DOX **5** or **6**) or red (for Dextran -A-647 or EGF A-647) channel was quantified as the average fluorescence intensity per pixels I_{cell} in live cells minus the average intensity I_{bg} of the background to give average fluorescence intensity in cells I_{CAX}. Relative cell viability (*RV*) was calculated as the count of Hoechst 33342 stained cells normalized to the count of Hoechst 33342 stained cells in absence of inhibitor. The number of detected cells in the absence of an inhibitor was $\approx 10^3$ after treatment with LUVs ⊃ DOX **5** or **6**.

8.3. Results

8.3.1. With TMU inhibitors

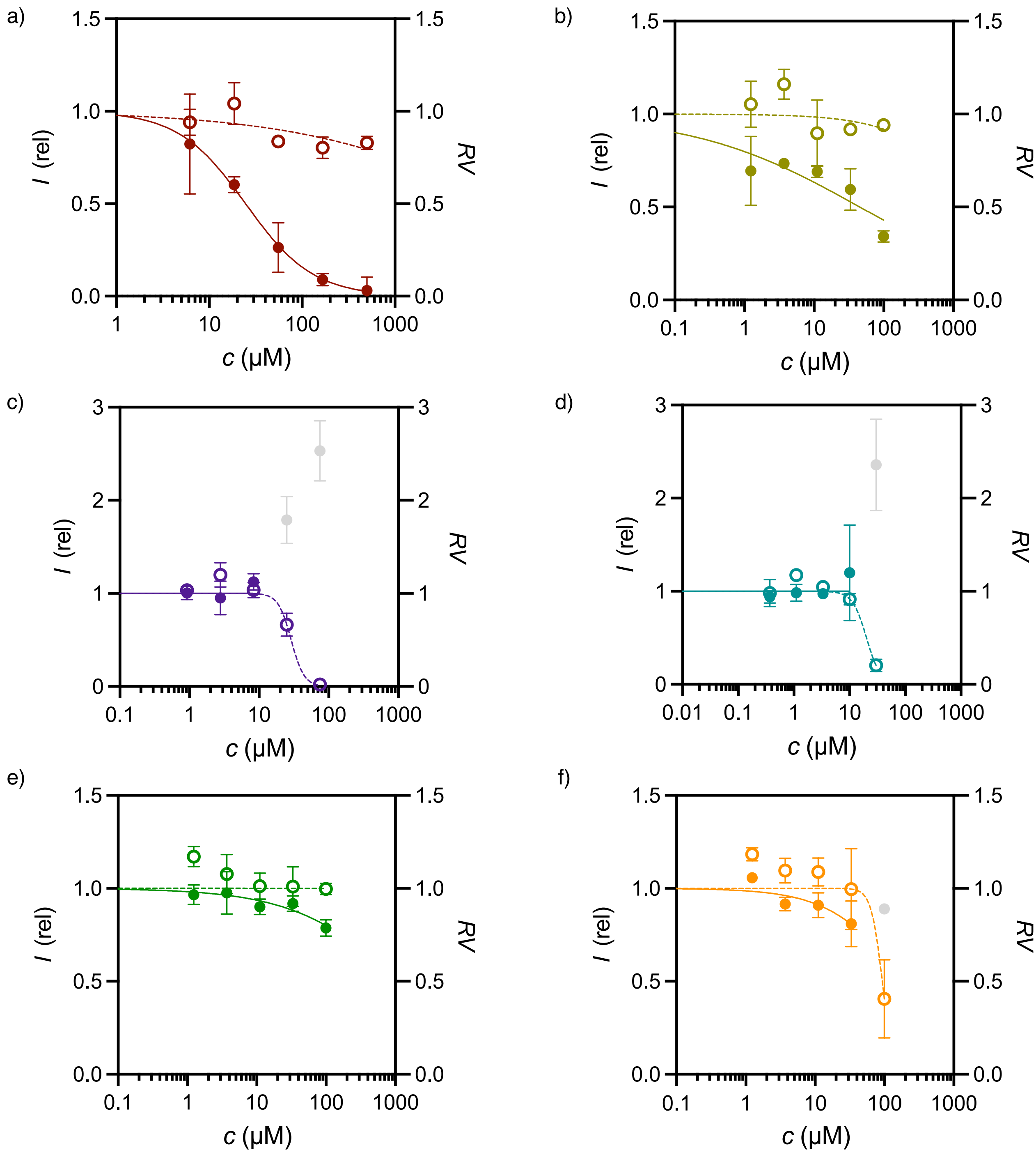

Fig. S26 Relative fluorescence intensity *I* (rel) ± SD (filled symbols) of DSPsC LUVs **5** ⊃ DOX (75 μM) in HK cells and relative viability *RV* ± SD (empty symbols) as a function of the concentration of (a) **7**, (b) **8**, (c) **9**, (d) **10**, (e) **23**, and (f) **24**. Grey symbols represent the data points excluded from the curve fit.

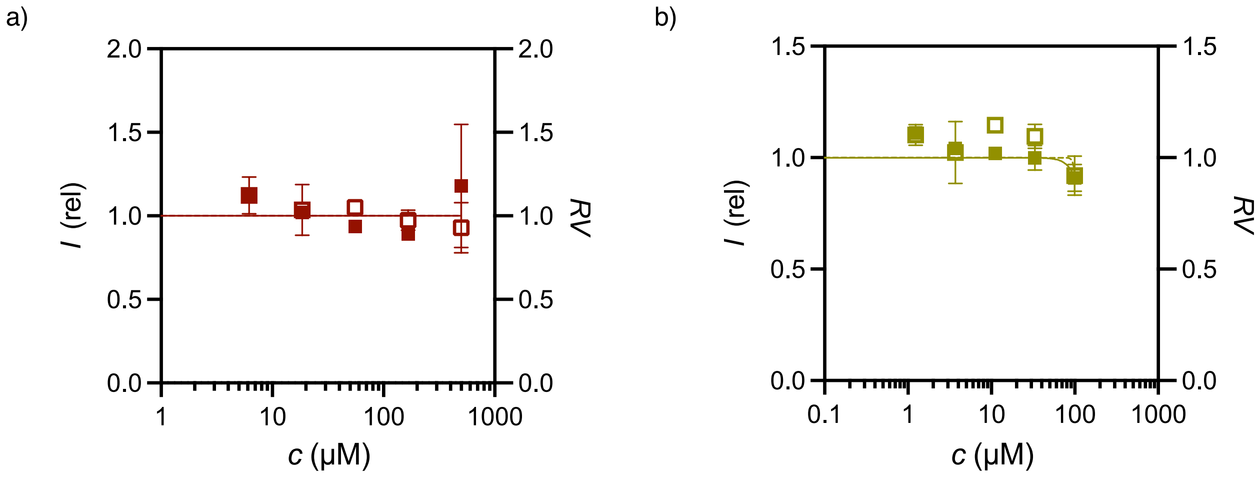

Fig. S27 Relative fluorescence intensity *I* (rel) ± SD (filled symbols) of DSPC LUVs **6** ⊃ DOX (75 μM) in HK cells and relative viability *RV* ± SD (empty symbols) as a function of the concentration of (a) **7** and (b) **8**.

Table S3 Dependence of cellular uptake of LUVs and cell viability in HK cells on the concentration of CAX inhibitors under pre-incubation conditions[a].

Entry	LUVs	Inhibitor	MIC (μM)[b]	IC_{50} (μM)[c]	*n* (IC_{50})[d]	RV_{50} (μM)[e]
1	**5**	**7**	6	25 ± 4	1.2 ± 0.2	>500
2	**5**	**8**	55	-	-	>100
3	**5**	**9**	-	-	-	~30
4	**5**	**10**	-	-	-	~20
5	**5**	**23**	<1	45 ± 20	0.4 ± 0.1	>100
6	**5**	**24**	25	>33	-	95 ± 30
7	**6**	**7**	-	-	-	>500
8	**6**	**8**	-	-	-	>100

[a]Results from dose-response curves in Figures S26-S27. [b]Concentration needed to reach 15% inhibition. [c]Concentration needed to reach 50% inhibition. [d]Hill coefficient for inhibition of cellular uptake. [e]Concentration needed to lower relative viability (RV) by 50%.

8.3.2. With endocytosis inhibitors

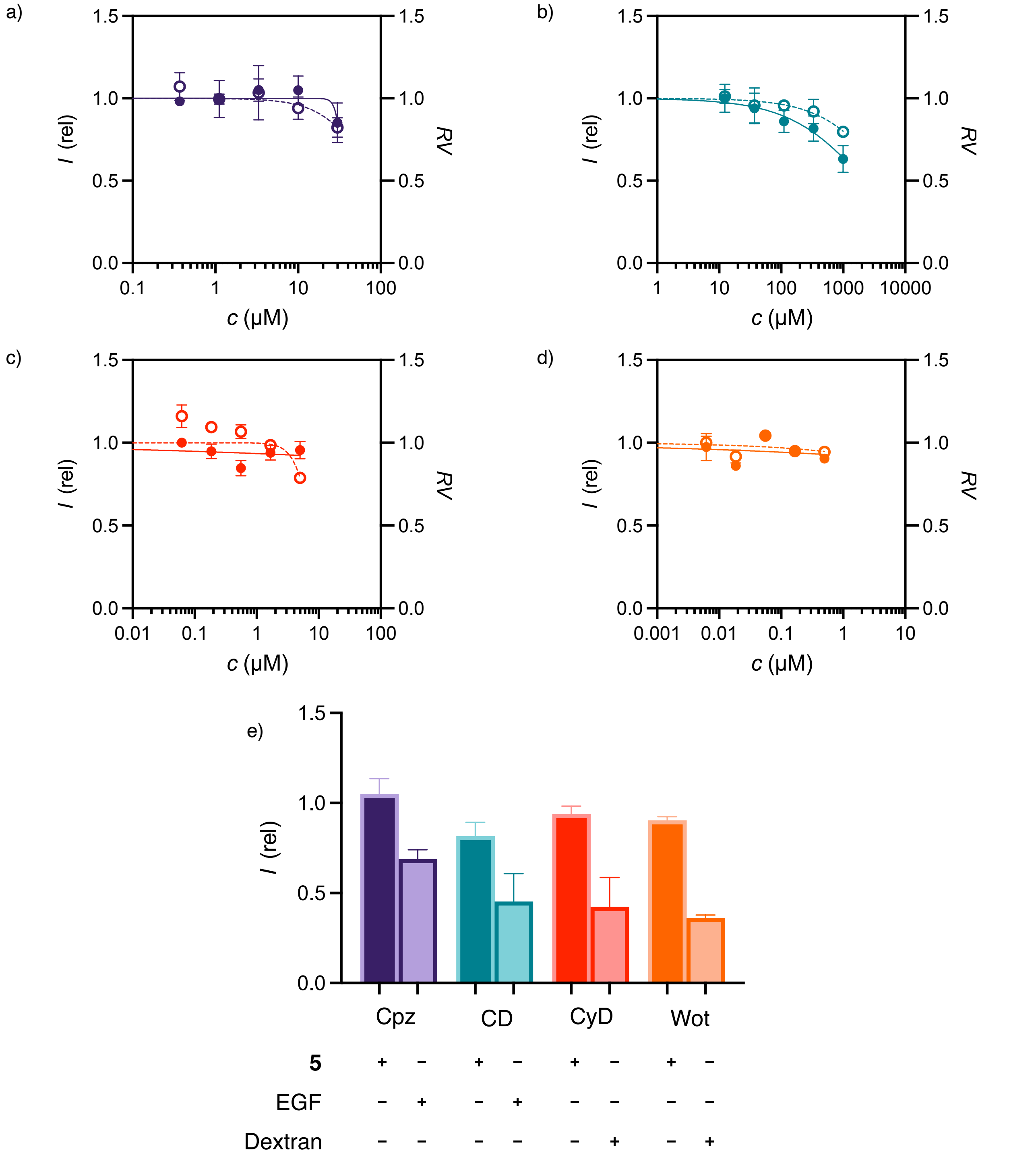

Fig. S28 Relative fluorescence intensity *I* (rel) ± SD (filled symbols) of DSPSC LUVs **5** ⊃ DOX (75 μM) in HK cells and relative viability *RV* ± SD (empty symbols) as a function of the concentration of (a) chlorpromazine (Cpz, 30 μM), (b) methyl-β-cyclodextrin (CD, 1000 μM), (c) cytochalasin D (CyD, 5 μM), (d) wortmannin (Wot, 0.5 μM). (e) Comparison of inhibition of DSPSC LUVs **5** ⊃

DOX and Epidermal growth factor (EGF, 200 ng/mL) or Dextran (2 mg/mL) with Cpz (10 µM), CD (333 µM), CyD (1.7 µM), and Wot (0.5 µM).

9. Evaluation of LUVs uptake by FLIM

9.1. Fluorescence lifetime determination by FLIM imaging of LUVs

E4P-Flipper **12** (1.7 µM) was embedded in DSPC LUVs **6**, DSP$_S$C LUVs **5**, or DOPC LUVs **38** (0.25 mM lipid, 5 mM HEPES, 172 mM NaCl, pH 7.4) as described in 5.1. The mixtures were placed in an 18-well plate, and the FLIM images were acquired using Leica Stellaris 8 Falcon with a 63X oil-immersion lens at room temperature. The excitation was at 488 nm (20 MHz, white laser), and the fluorescence was collected between 550 and 650 nm. The lifetimes were estimated by manually selecting the center of photon clouds in phasor plots.[S16]

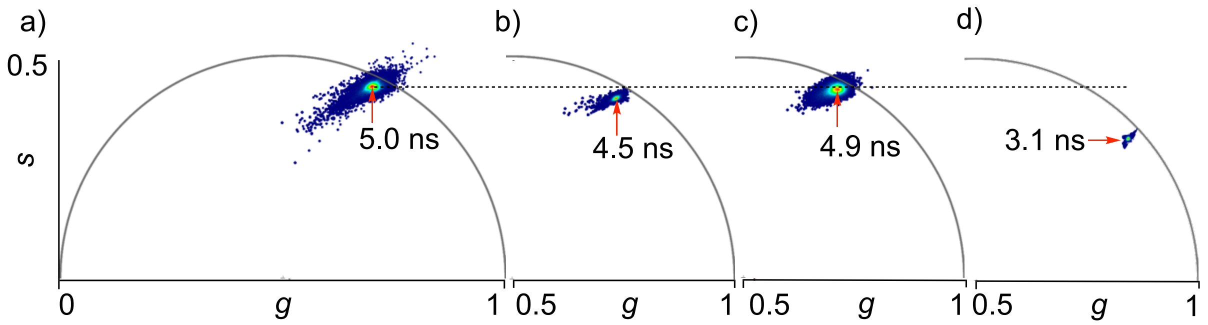

Fig. S29 Phasor plots of FLIM images in Figure 4d. E4P-Flipper **12** in a) DSPC LUVs **6**, b) DSP$_S$C LUVs **5**, c) activated DSP$_S$C LUVs **16c**, or d) DOPC LUVs **38**.

9.2. Imaging uptake of LUVs in HK cells by FLIM

HK cells were plated and grown on 35 mm glass-bottomed dishes, as described in section 6. Cells were rinsed (3 × 1 mL PBS, 3 × 1 mL L-15) and incubated with E4P-Flipper **12** (0.5 µM in 1.0 mL L-15), or LUVs **5**, or **6** (125 µM) containing E4P-Flipper **12** (0.7 mol%) in L-15 (1.0 mL) for 2 h at 37 °C under 5% CO_2. Afterward, cells were rinsed (3 × 1 mL DPBS, 3 × 1 mL L-15), kept in L-15 (≈0.5 mL), and covered with a glass coverslip. The glass bottom dish was then flipped upside-

down (inverted mode) for FLIM imaging using Leica SP8DIVE Falcon with 63X oil-immersion lens at 37 °C. The excitation laser was at 488 nm (20 MHz, white laser), and the fluorescence was collected between 550 and 650 nm. Laser power (LP) and brightness of images were individually adjusted.

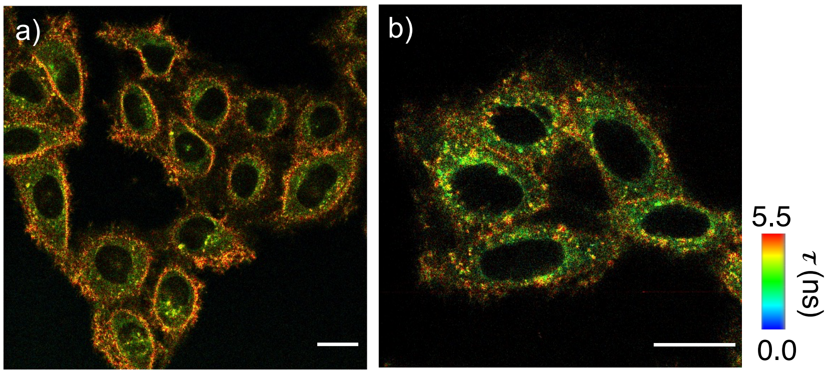

Fig. S30 Fast FLIM images of (a) E4P-Flipper **12** alone (LP 1%), and (b) embedded in DSPsC LUVs **5** (LP 5%) after 2 h incubation with HK cells. Compare Figures 4a, b. Scale bars = 20 µm.

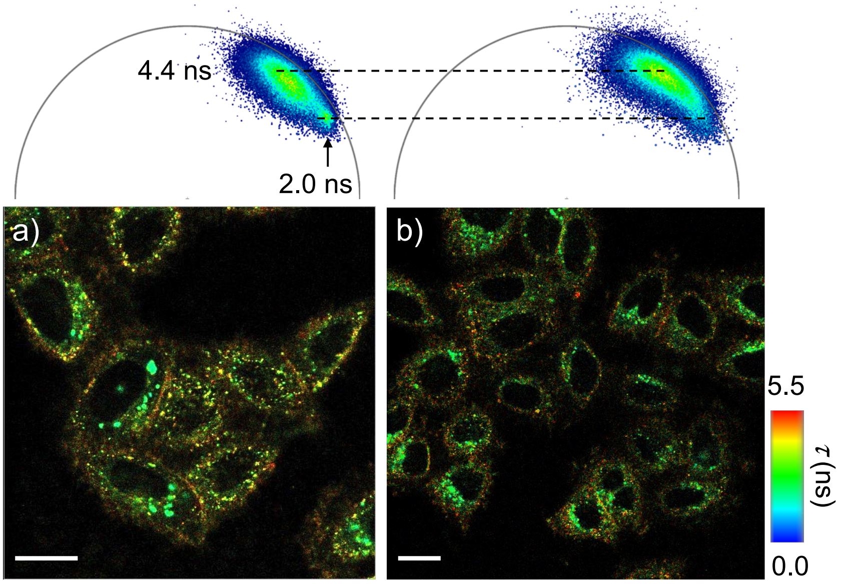

Fig. S31 Fast FLIM images and corresponding Phasor plots of E4P-Flipper **12** embedded in a) DSPsC LUVs **5**, and b) **5** activated with **15**, after 2 h of incubation with HeLa-Kyoto cells. Compare Figure 4d. LP = 5%. Scale bars = 20 µm.

10. Co-localization

HK cells were seeded at 8 × 10^4 cells/well on 35 mm glass-bottomed dishes (MatTek Corporation) and cultured overnight.

For BODIPY™ 493/503 tracker. After removing the medium, HK cells were washed with PBS (3 x 1 mL) and with L-15 medium (3 x 1 mL) before being treated with DSP_SC: 16:0 Liss Rhod PE **35**, or DSPC: 16:0 Liss Rhod PE **36** (75 µM, in 1.0 mL Leibovitz's L-15 medium). The cells were incubated for 2 h at 37 °C, then the media was removed by aspiration. Cells were washed with PBS (3 x 1 mL) and with L-15 medium (3 x 1 mL) before being treated with BODIPY™ 493/503 tracker (10 µM, in 1.0 mL Leibovitz's L-15 medium) for 10 min. The cells were kept in L-15 medium during the microscope experiment. Distribution of fluorescence was analyzed without fixing using a confocal laser scanning microscope (Leica SP8) equipped with 63X oil immersion objective lens. A laser was used as light source (20% laser power) with excitation wavelength 552 nm and emission 580-630 nm (Leica HyD™ detector) and another as light source (0.4% laser power) with excitation wavelength 488 nm and emission 500-540 nm (Leica PMT™ detector).

For LysoTracker™ Red DND-99. After removing the medium, HK cells were incubated with E4P-Flipper **12** in DSP_SC LUVs **5** or DSPC LUVs **6** for 2 h, rinsed (3 × 1 mL PBS, 3 × 1 mL L-15), and kept in L-15 (≈0.5 mL) containing LysoTracker™ Red (0.1 µM). CLSM images were acquired after 5–20 min of incubation without rinsing using Leica SP8DIVE Falcon in inverted mode with 63X oil-immersion lens at 37 °C. Images were acquired consecutively at 550–600 nm upon excitation at 488 nm for E4P-Flipper **12**, and at 600–700 nm upon excitation at 561 nm for LysoTracker.

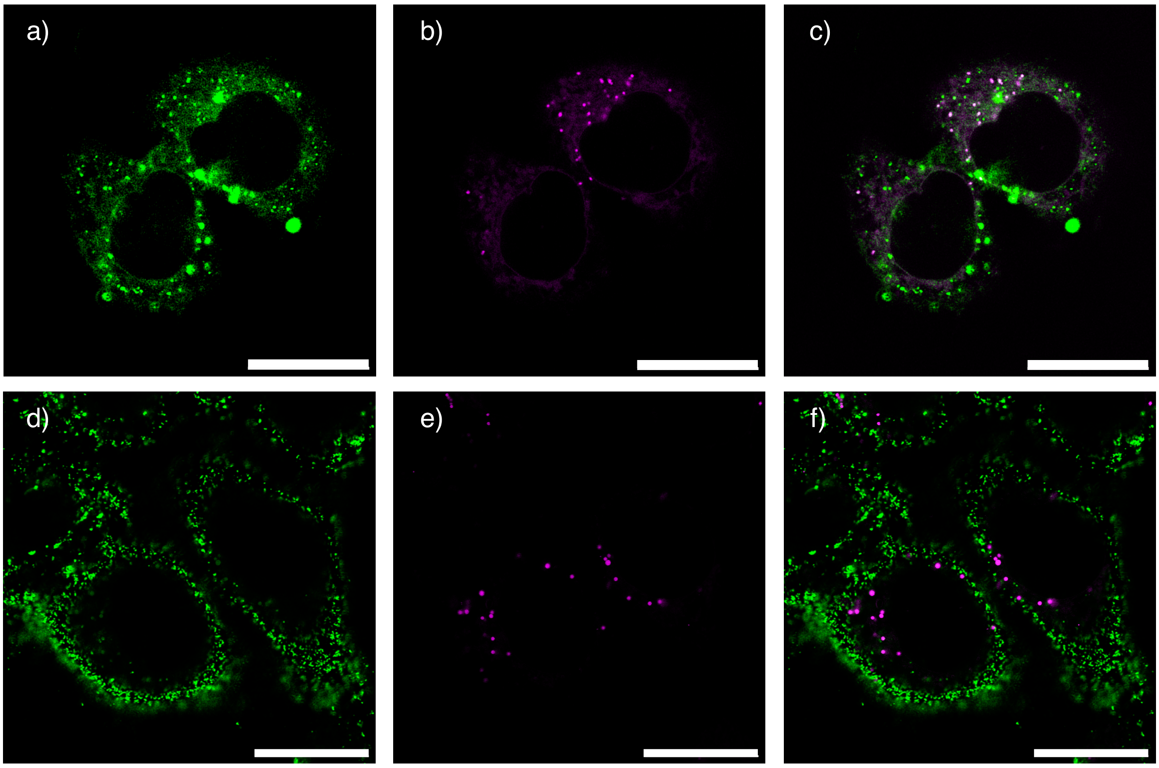

Fig. S32 CLSM images of (a–c) DSP$_S$C: 16:0 Liss Rhod PE LUVs **35** or (d–f) DSPC: 16:0 Liss Rhod PE LUVs **36** after 2 h incubation with HK cells detected at 580-630 nm for 16:0 Liss Rhod PE LUVs (a, d), at 500-540 nm for BODIPY™ 493/503 (b, e), and overlay (c, f). Scale bars = 20 µm. Poor overlap in staining of DSPC: 16:0 Liss Rhod PE LUVs **36** and BODIPY™ 493/503 in DSPC-LUVs (d–f) demonstrates the absence of bleed-through under these conditions.

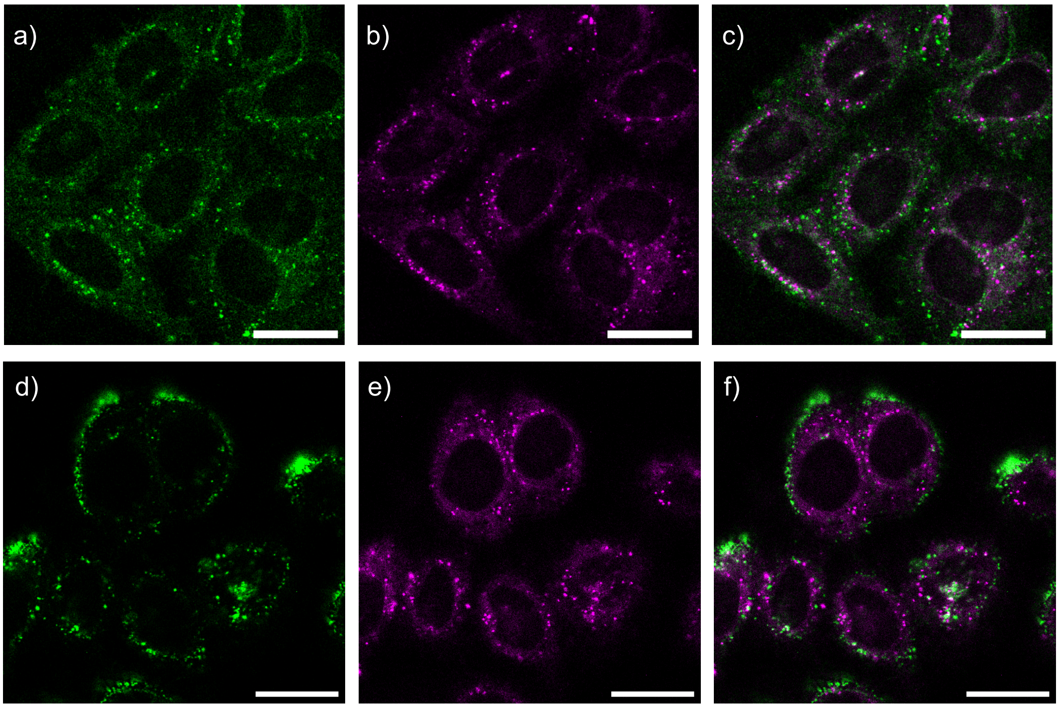

Fig. S33 CLSM images E4P-Flipper **12** in (a–c) DSP$_S$C LUVs **5** or (d–f) DSPC LUVs **6** after 2 h incubation with HK cells detected at 550-600 nm for E4P-Flipper **12** (a, d), at 600-700 nm for LysoTracker (b, e), and overlay (c, f). Scale bars = 20 µm. Poor overlap in staining of **12** and LysoTracker in DSPC-LUVs (d–f) demonstrates the absence of bleed-through under these conditions.

11. Supplementary references

S1 J. Bouffard, F. Coelho, N. Sakai and S. Matile, *Angew. Chem. Int. Ed.*, 2023, **62**, e202313931.

S2 O. Kozlov, E. Horáková, S. Rademacherová, D. Maliňák, R. Andrýs, E. Prchalová and M. Lísa, *Anal. Chem.*, 2023, **95**, 5109–5116.

S3 C. A. H. Prata, X.-X. Zhang, D. Luo, T. J. McIntosh, P. Barthelemy and M. W. Grinstaff, *Bioconjugate Chem.*, 2008, **19**, 418–420.

S4 I. Vasilenko, B. de Kruijff and A. J. Verkleij, *Biochim. Biophys. Acta*, 1982, **685**, 144–152.

S5 G. Gasparini, G. Sargsyan, E.-K. Bang, N. Sakai and S. Matile, *Angew. Chem. Int. Ed.*, 2015, **54**, 7328–7331.

S6 L. Zong, E. Bartolami, D. Abegg, A. Adibekian, N. Sakai and S. Matile, *ACS Cent. Sci.*, 2017, **3**, 449–453.

S7 Y. Cheng, L. Zong, J. López-Andarias, E. Bartolami, Y. Okamoto, T. R. Ward, N. Sakai and S. Matile, *Angew. Chem. Int. Ed.*, 2019, **58**, 9522–9526.

S8 I. Shybeka, J. R. J. Maynard, S. Saidjalolov, D. Moreau, N. Sakai and S. Matile, *Angew. Chem. Int. Ed.*, 2022, **61**, e202213433.

S9 F. Bayard and S. Matile, *Helv. Chim. Acta*, 2024, **107**, e202400062.

S10 Q. Laurent, R. Martinent, D. Moreau, N. Winssinger, N. Sakai and S. Matile, *Angew. Chem. Int. Ed.*, 2021, **60**, 19102–19106.

S11 K. K. P. Pamungkas, I. Fureraj, L. Assies, N. Sakai, V. Mercier, X.-X. Chen, E. Vauthey and S. Matile, *Agew. Chem. Int. Ed.*, 2024, **63**, e202406204.

S12 G. Niu, B. Cogburn and J. Hughes, in *Cancer Nanotechnology: Methods and Protocols*, eds. S. R. Grobmyer and B. M. Moudgil, Humana Press, Totowa, NJ, 2010, pp. 211–219.

S13 M. C. Smith, R. M. Crist, J. D. Clogston and S. E. McNeil, *Anal. Bioanal. Chem.*, 2017, **409**, 5779–5787.

S14 J. López-Andarias, K. Eblighatian, Q. T. L. Pasquer, L. Assies, N. Sakai, S. Hoogendoorn and S. Matile, *Angew. Chem. Int. Ed.*, 2022, **61**, e202113163.

S15 S. Saidjalolov, X.-X. Chen, J. Moreno, M. Cognet, L. Wong-Dilworth, F. Bottanelli, N. Sakai and S. Matile, *JACS Au*, 2024, **4**, 3759–3765.

S16 S. Ranjit, L. Malacrida, D. M. Jameson and E. Gratton, *Nat. Protoc.*, 2018, **13**, 1979–2004.

12. NMR spectra

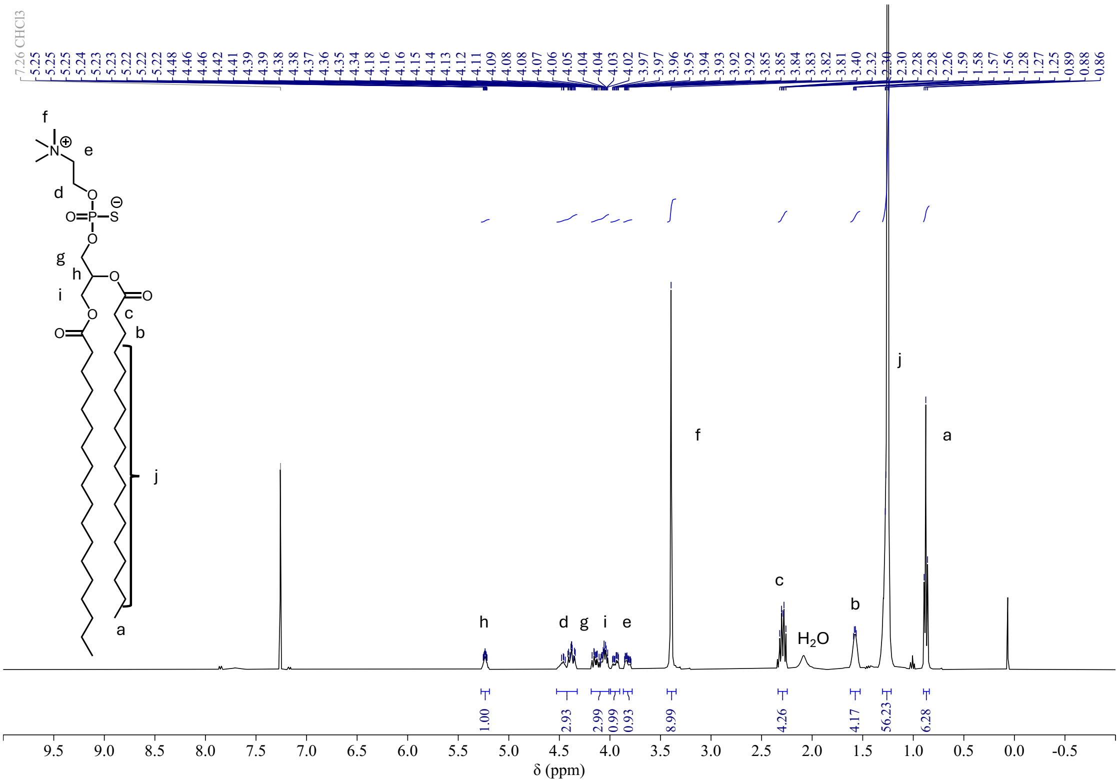

Fig. S34 400 MHz ^{1}H NMR spectrum of **3** in $CDCl_3$.

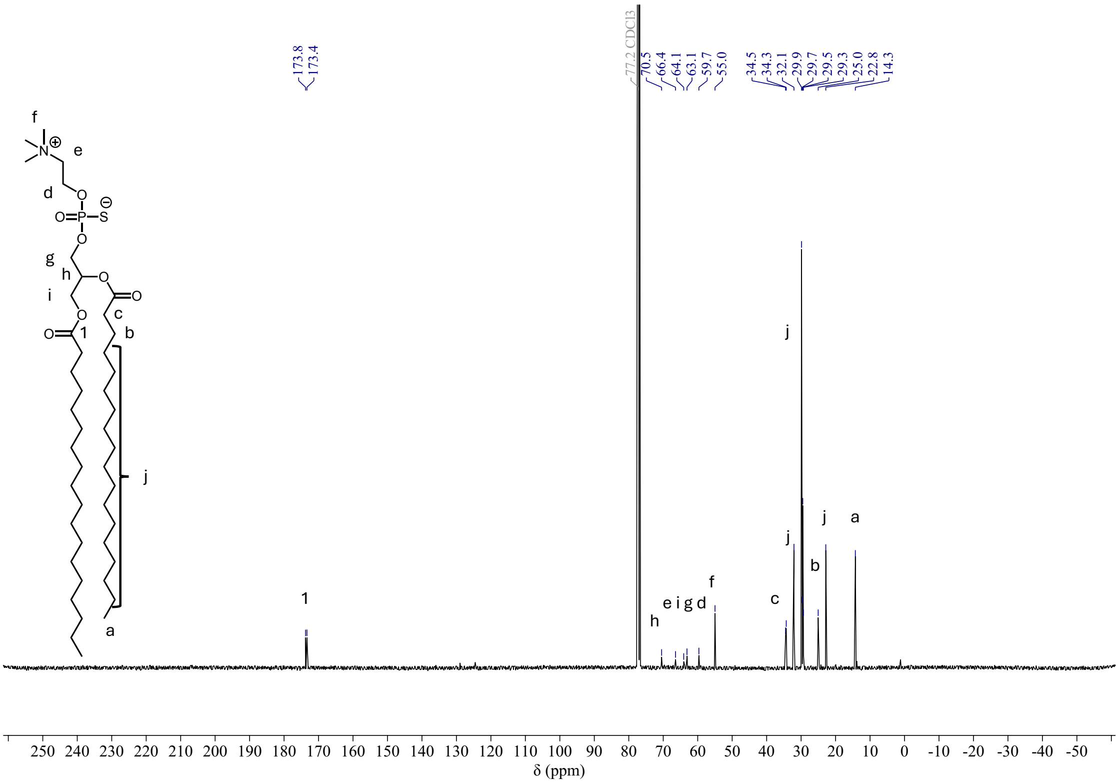

Fig. S35 101 MHz ^{13}C NMR spectrum of **3** in $CDCl_3$.

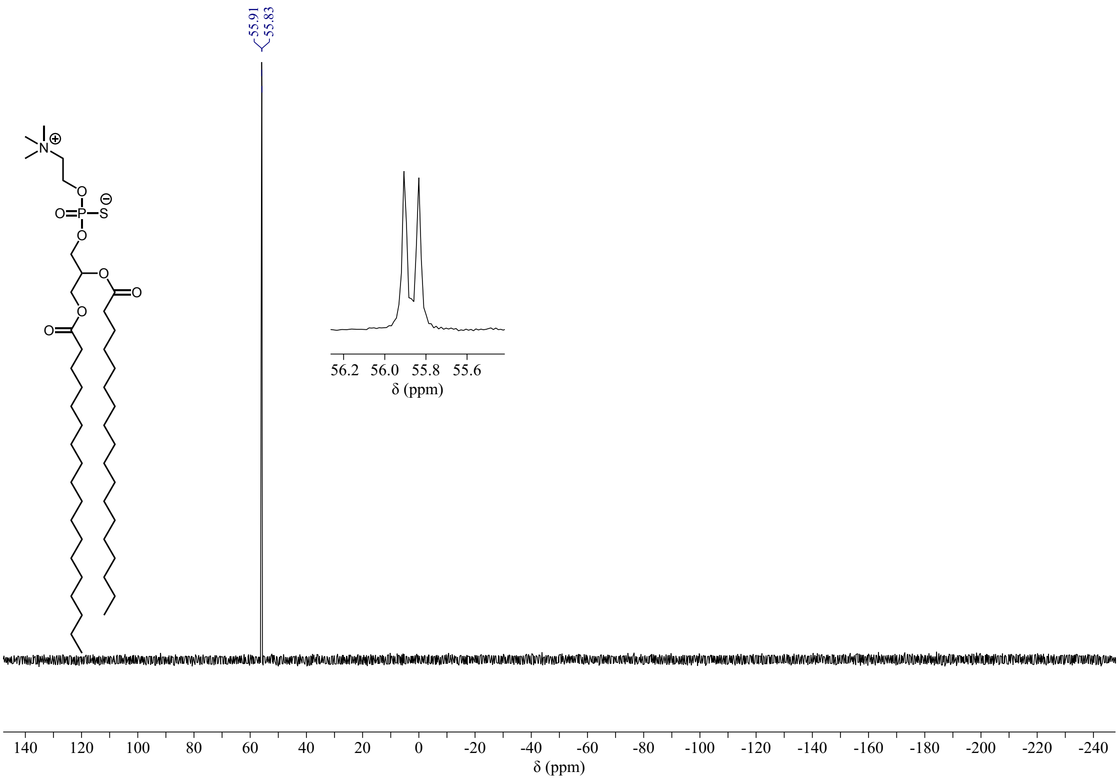

Fig. S36 162 MHz ^{31}P NMR spectrum of **3** in $CDCl_3$.

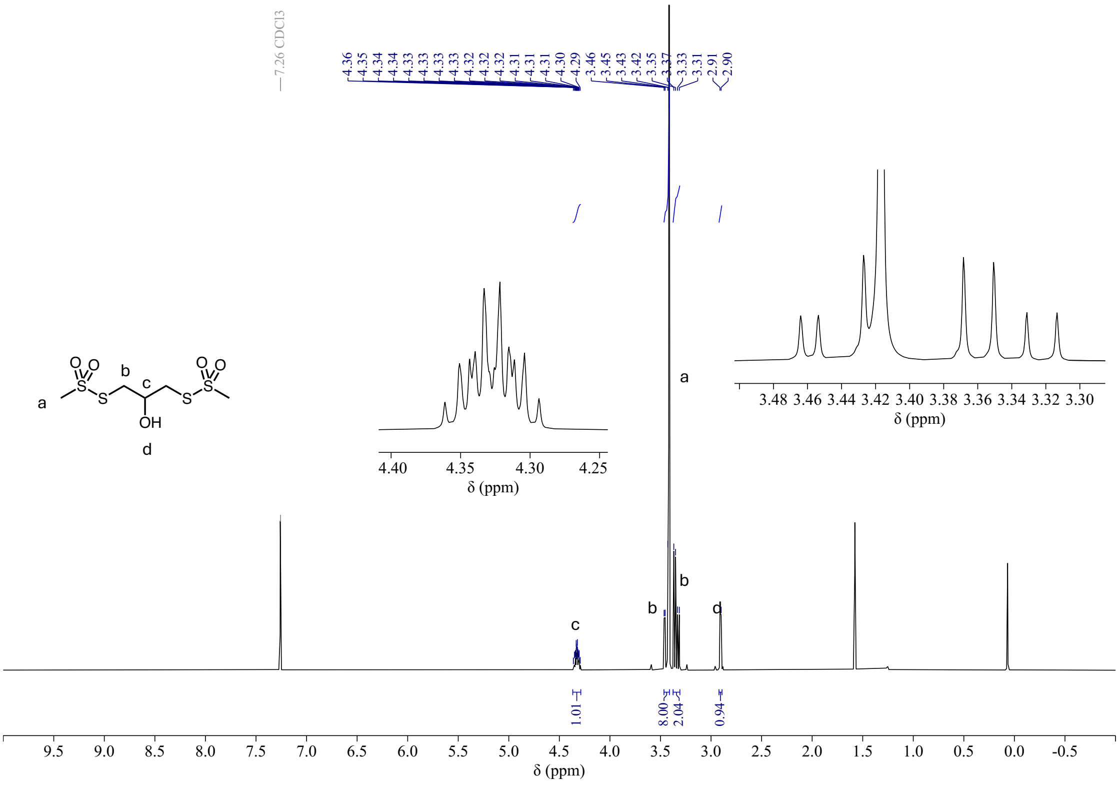

Fig. S37 400 MHz ^{1}H NMR spectrum of **15** in $CDCl_3$.

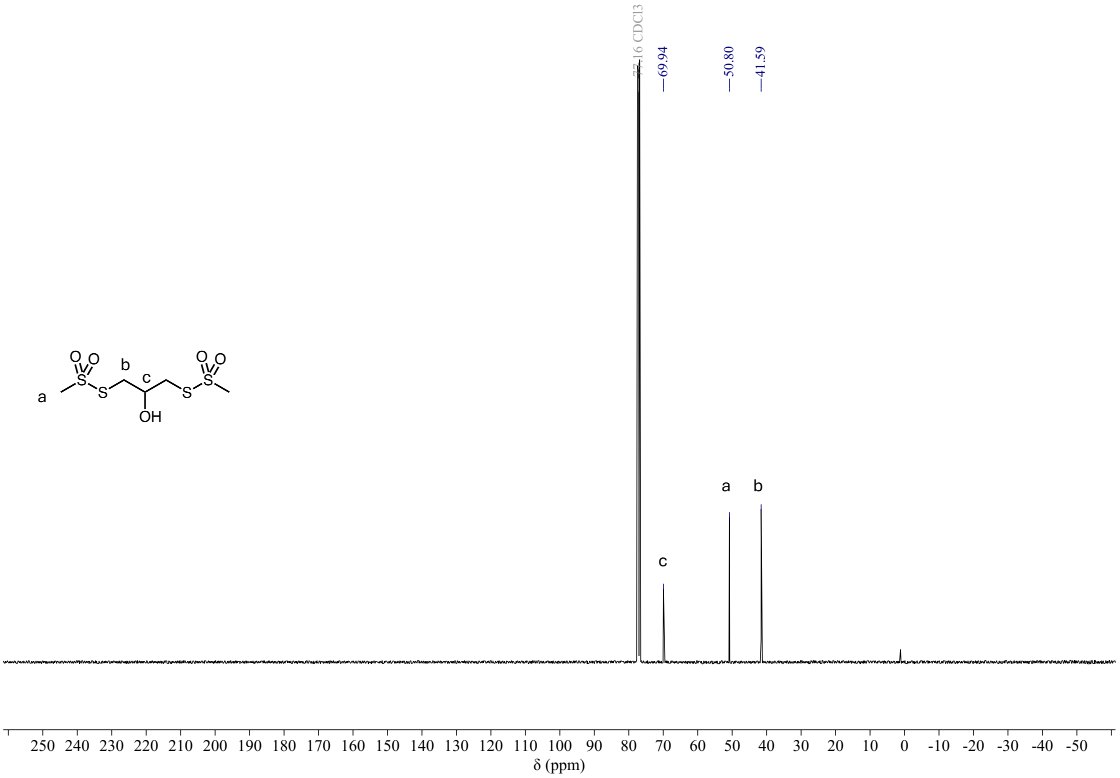

Fig. S38 101 MHz ^{13}C NMR spectrum of **15** in $CDCl_3$.
